# Clinical Outcomes With Medium Cut-Off Versus High-Flux Hemodialysis Membranes: A Systematic Review and Meta-Analysis

**DOI:** 10.1177/20543581211067087

**Published:** 2022-01-21

**Authors:** Maryam Kandi, Romina Brignardello-Petersen, Rachel Couban, Celina Wu, Gihad Nesrallah

**Affiliations:** 1 Department of Health Research Methods, Evidence & Impact, McMaster University, Hamilton, ON, Canada; 2 University of Toronto, ON, Canada; 3 Nephrology Program, Humber River Hospital, Toronto, ON, Canada

**Keywords:** Theranova, medium cut-off, expanded hemodialysis, large middle molecules, meta-analysis, dialysis outcomes

## Abstract

**Background:**

A novel medium cut-off (MCO) dialyzer (Theranova, Baxter Healthcare, Deerfield, IL, USA) enhances large middle molecule clearance while retaining selectivity for molecules >45 000 Da.

**Objective:**

We undertook a systematic review and meta-analysis evaluating clinical outcomes with MCO vs high-flux membranes.

**Methods:**

We searched MEDLINE, EMBASE, CINAHL, Cochrane Library, and Web of Science through July 2020, and gray literature sources from 2017. We included randomized (RS) and nonrandomized studies (NRS) comparing MCO and high-flux membranes in adults receiving maintenance hemodialysis. Pairs of reviewers performed study selection, data extraction, and risk of bias assessment in duplicate. We conducted random-effects pairwise meta-analyses to pool results across studies and used the Grading of Recommendations Assessment, Development and Evaluation approach to assess evidence certainty.

**Results:**

We identified 22 eligible studies (6 RS, 16 NRS; N = 1811 patients; patient-years = 1546). The MCO dialyzer improved (estimate; 95% confidence interval [CI]; certainty rating) quality of life (mean difference [MD] = 16.7/100 points; 6.9 to 26.4; moderate), Kidney Disease Quality of Life Instrument (KDQOL) subscales—burden (MD = 4.0; 1.1 to 6.9; moderate) and effects (MD = 5.4; 3.2 to 7.6; moderate), pruritus (MD = −4.4; −7.1 to −1.7; moderate), recovery time (MD = −420 minutes; −541 to −299; high), and restless legs syndrome (odds ratio = 0.39; 0.29 to 0.53; moderate). There was little to no difference in all-cause mortality (risk difference = −0.4%; −2.8 to 2.1; moderate) and serious adverse events (rate ratio = 0.63; 0.38 to 1.04; low). MCO dialysis reduced hospitalization (rate ratio = 0.48; 0.27 to 0.84; low), infection (rate ratio = 0.38; 0.17 to 0.85; moderate), hospitalization days (MD = −1.5 days; 95% CI, −2.22 to −0.78; moderate), erythropoiesis resistance index (MD = −2.92 U/kg/week/g/L; 95% CI, −4.25 to −1.6; moderate) and cumulative iron use over 12 weeks (MD = −293 mg; 95% CI, −368 to −218; moderate). We found with low certainty that MCO dialysis had little to no effect on KDQOL symptoms/problem list, pain, and physical health and moderate certainty that MCO dialysis likely has no effect on the KDQOL mental health composite.

**Conclusions:**

We found with predominantly moderate certainty that the MCO dialyzer improves several patient-important outcomes with no apparent risks or harms. More definitive studies are needed to better quantify the effects of MCO membranes on mortality, hospitalization, and other rare events.

## Abrégé

**Contexte:**

Un nouveau dialyseur MCO (Medium cut-off) (Theranova, Baxter Healthcare, Deerfield, IL, É.-U.) améliore la clairance des molécules importantes de taille moyenne tout en maintenant la sélectivité des molécules de plus de 45 000 Da.

**Objectifs:**

Nous avons entrepris une revue systématique et une méta-analyse évaluant les résultats cliniques des membranes MCO par rapport aux membranes à perméabilité élevée.

**Méthodologie:**

Nous avons effectué des recherches dans MEDLINE, embase, CINAHL, Cochrane Library et Web of Science jusqu’en juillet 2020, et dans des sources de littérature grise de 2017. Nous avons inclus les études randomisées (ÉR) et non randomisées (ÉNR) comparant les membranes MCO et les membranes à perméabilité élevée chez les adultes recevant une hémodialyse d’entretien. Des paires de réviseurs ont procédé à la sélection des études, à l’extraction des données et à l’évaluation du risque de biais en duplicata. Nous avons effectué des méta-analyses à effets aléatoires par paires pour regrouper les résultats des différentes études, puis nous avons employé la méthodologie GRADE (Grading of Recommendations Assessment, Development and Evaluation) pour évaluer la certitude des preuves.

**Résultats:**

Nous avons répertorié 22 études admissibles (6 ÉR, 16 ÉNR ; n=1811 patients; 1 546 années-patients). Le dialyseur MCO a amélioré (estimation; IC à 95 %; évaluation de la certitude) la qualité de vie (différence moyenne [DM] = 16,7/100 points; 6,9 à 26,4; modérée), les sous-échelles KDQOL — le fardeau de la maladie (DM = 4,0; 1,1 à 6,9; modérée), les effets (DM = 5,4; 3,2 à 7,6; modérée), le prurit (DM = -4,4; -7,1 à -1,7; modérée), le temps de récupération (DM = -420 minutes; -541 à -299; élevée) et le syndrome des jambes sans repos (rapport de cotes = 0,39; 0,29 à 0,53; modéré). On a noté peu ou pas de différence pour la mortalité toutes causes confondues (risque différentiel = -0,4 %; -2,8 à 2,1; modérée) et les événements indésirables graves (rapport des taux = 0,63; 0,38 à 1,04; faible). La dialyse par MCO a réduit les hospitalisations (rapport des taux = 0,48; 0,27 à 0,84; faible), les infections (rapport des taux = 0,38; 0,17 à 0,85; modérée), la durée des hospitalisations (DM = -1,5 jour; -2,22 à -0,78; modérée), l’indice de résistance à l’érythropoïèse (DM = -2,92 U/kg/semaine/g/L; -4,25 à -1,6; modérée) et l’utilisation cumulative de fer sur 12 semaines (DM = -293 mg; -368 à -218; modérée). Nous avons constaté, avec peu de certitude, que la dialyse MCO n’avait que peu ou pas d’effet sur les symptômes/problèmes liés à la KDQOL, de même que sur la douleur et la santé physique. Et nous avons constaté, avec une certitude modérée, que la dialyse MCO n’avait probablement aucun effet sur le composite de santé mentale de la KDQOL.

**Conclusion:**

Nous avons constaté avec une certitude principalement modérée que le dialyseur MCO améliorait plusieurs résultats importants pour le patient sans risques ou préjudices apparents. Des études plus définitives sont nécessaires afin de mieux quantifier les effets des membranes MCO sur le taux de mortalité, les hospitalisations et les autres événements rares.

## Introduction

Suboptimal removal of larger middle molecules with hemodialysis contributes to the persistence of the uremic state and its complications. While convective therapies enhance the elimination of large middle molecules, they have been difficult to scale, while high cut-off membranes remove desirable molecules including albumin.

A novel medium cut-off (MCO) membrane (Theranova 400/500, Baxter Healthcare, Deerfield, IL, USA) removes large middle molecules while excluding those >45 kDa,^1^ using larger pores within a narrow diameter distribution. By optimizing this “cut-off” threshold, MCO membranes can maximize larger uremic solute clearance while minimizing unintended solute losses and could thereby significantly impact outcomes. We conducted a systematic review and meta-analysis on the comparative effects of MCO vs high-flux membranes for maintenance hemodialysis.

## Methods

### Protocol and Registration

Our registered protocol (PROSPERO: CRD42020204636) and amendments are in Online Appendix A. We prepared this manuscript in accordance with the PRISMA (Preferred Reporting Items for Systematic Reviews and Meta-Analyses) checklist. We present abbreviated methods with further details in Online Appendix B.

### Eligibility Criteria

We included randomized and nonrandomized studies published in any language from 2015 (first year that MCO dialyzers were commercially available), which enrolled adult outpatients receiving maintenance hemodialysis with an MCO dialyzer or related prototypes. We excluded studies of high cut-off and “super high-flux” membranes. Eligible comparators were high-flux membranes used for hemodialysis; convective therapies were excluded. Prespecified outcomes are in Online Appendix B; categories included major clinical events, patient-reported outcomes (PROs), drug utilization, and safety.

### Information Sources

We searched MEDLINE, EMBASE, CINAHL, the Cochrane Library, and Web of Science through July 2020. Gray literature sources included abstracts from prespecified major conferences.

### Search

Search concepts used by our information specialist (R.C.) were hemodialysis and MCO membranes. Synonyms for each concept were combined using the OR operator and then the concepts were combined using the AND operator. The search strategy is in Online Appendix C.

### Study Selection

We used EndNote X9.3 for de-duplication and DistillerSR for title and abstract and full-text screening by 2 reviewers.

### Data Collection Process

Reviewers extracted data independently into standard forms with verification by second reviewer.

### Data Items

Details are in Online Appendices A and B. We extracted counts of patients with clinical events (eg, infection, hospitalization) to avoid double counting and extracted total counts of events where patient-level details were unavailable.

### Risk of Bias in Individual Studies

Two reviewers independently used the Cochrane Risk of Bias tools version 2 for randomized studies,^2^ crossover trials,^3^ and the ROBINS-I tool for nonrandomized studies.^4^ ROBINS-I includes 7 domains that compare each nonrandomized study to an “ideal” pragmatic trial, enabling direct comparisons of the certainty of evidence arising from randomized and nonrandomized studies for a given outcome.

We anticipated significant potential carryover effects such as a sustained reduction in large middle-molecule concentrations after treatment with the MCO dialyzer. However, as this effect would have biased effect estimates toward the null, we did not rate down for risk of bias based on the duration of washout periods in crossover trials.

### Summary Measures

For continuous outcomes, we extracted change scores and corresponding standard errors (SEs) and used *P* values to impute the SE for change where required, then calculated the mean difference between groups, and standardized mean differences (SMD) where units of measure differed. We used final values when change scores were not reported. For binary outcomes, we considered the patient as the unit of analysis and calculated relative risk and rate ratios when counts of events were reported. We calculated odds ratios for outcomes measured cross-sectionally.

### Synthesis of Results

For each outcome, we used generic inverse variance to pool results across studies separately for randomized and nonrandomized studies, using RevMan 5.4. We used random-effects models, using fixed-effects models to avoid overweighting when pooling 2 studies. A blinded external collaborator grouped PRO measures for meta-analysis to guard against potential bias.^5^ We used the *I*^2^ statistic to measure heterogeneity.

### Additional Analyses

Where intent-to-treat analyses were potentially biased by high attrition rates, we performed sensitivity analyses using per-protocol data. We also performed sensitivity analyses in which we excluded abstracts from the pooled estimates, where applicable.

### Certainty of Evidence

We assessed the certainty of evidence separately for each outcome using Grading of Recommendations Assessment, Development and Evaluation (GRADE) and summarized these assessments in a Summary of Findings Table using GRADEpro: https://gdt.gradepro.org.^6^ Certainty was rated as very low, low, moderate, or high. Effect estimates for randomized and nonrandomized studies started with high certainty and were downgraded 1 or 2 levels for risk of bias,^7,8^ inconsistency,^9^ indirectness,^10^ imprecision,^11^ or publication bias.^12^ We appraised certainty on an outcome-by-outcome basis, considering the specific studies contributing to each effect estimate. In doing so, we considered the relative contribution (weight) of each study when rating the risk of bias across studies. We rated up for large effects, dose-response, and opposing residual confounding bias in nonrandomized studies.^13^ We assessed imprecision for dichotomous outcomes using nomograms for optimal information size. For continuous outcomes, we estimated optimal information size using sample size calculators for paired and unpaired comparisons as appropriate for the observed effect size, using β= 0.8 and α= 0.05.^11^ We calculated absolute treatment effects based on control event rates in studies included for each outcome.^14^ We used validated algorithms to produce informative qualitative statements describing review findings and used these phrases throughout this report (Table 2, column labeled “What Happens”).^15^

## Results

### Study Selection

**Figure 1.**
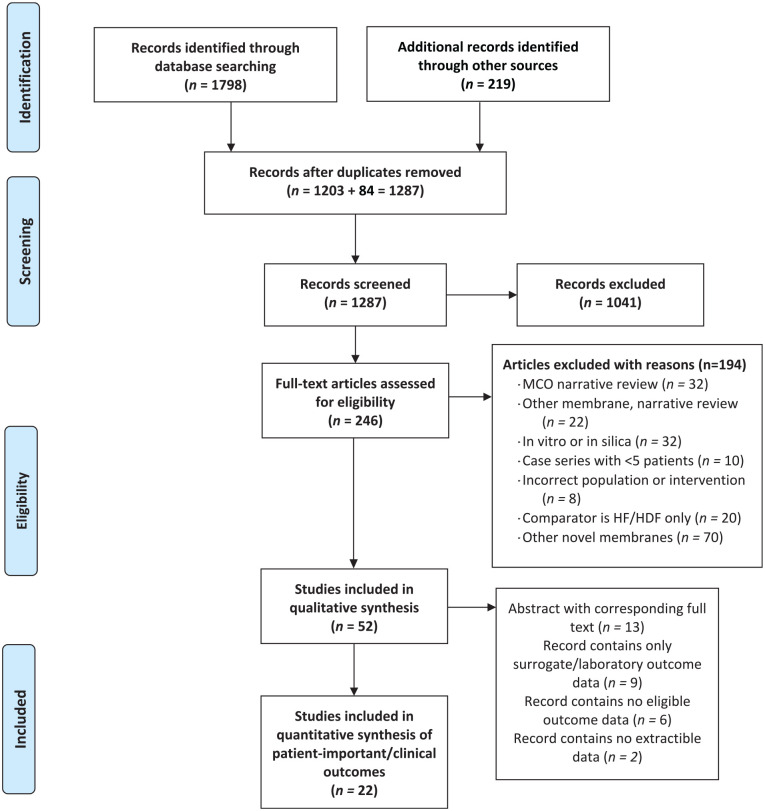
Study inclusion flow diagram.

We identified 52 eligible reports of 36 unique studies (Figure 1). Twenty-two studies reporting clinical outcomes were included in this report. Groupings of related citations are in Online Appendix D. We updated our review when 6 reports initially identified as abstracts or pre-prints were subsequently published as peer-reviewed full texts.^16-21^

### Study Characteristics

The 22 included studies comprised 6 randomized studies,^20-26^ including 2 parallel-arm^20-22^ and 4 crossover trials.^23-26^ Among the nonrandomized studies, 2 were cohort studies^27,28^ and the remainder used before-after^16-18,29-37^ or crossover designs.^38^ Six were abstracts,^29,30,32,34,37^ and the remaining 16 were full texts, one of which was a manuscript under review.^31^ Theranova was the only MCO membrane identified in our search. Details of patient, study design, and intervention characteristics are given in Table 1. Adult outpatients from diverse geographies underwent conventional hemodialysis with Theranova 400/500 or high-flux membranes, using standard anticoagulation protocols.

**Table 1.** Characteristics of Included Studies, Populations, and Interventions.

Author	Publication type	Country (number of centers)	Number of participants enrolled (number analyzed)	Follow-up (weeks)	Mean age ± SD (years)	% Male	Interventions	Reported outcomes
Randomized controlled trials								
Parallel arm studies								
Lim et al^20,22^	Full text	Korea (1)	50 (49)	12	I: 62.2 ± 13.7 C: 63.8 ± 15.2	66	I: Theranova 400 C: FxCordiax 80 or 60	Survival, QoL (KDQO)L, pruritus, adverse events, ERI, iron use, albumin (predialysis), MM
Weiner et al^21^	Full text	USA (21)	172 (130)	24	59 ± 13	39	I: Theranova 400 C: Elisio-17H	Survival, hospitalization, QoL (KDQOL, EQ-5D-5L), albumin (predialysis), MM
Randomized crossover trials								
Belmouaz et al^23^	Full text	France (1)	40 (40)	26	75.5 ± 9.9	70	I: Theranova 500 C: Elisio 21H	Survival, ERI, iron utilization, albumin RR, albumin (predialysis), MM
Santos et al^24^	Full text	Spain (1)	13 (13)	2	60.1 ± 4.6	92	I: Theranova 500 C: FxCordiax 80VR	Bleeding, extracorporeal circuit clotting, aPTT, anti-Xa
Sevinc et al^25^	Full text	Turkey (2)	52 (42)	26	56.4 (median)	58	I: Theranova 500 C: FxCordiax 80	Adverse events, albumin (predialysis), MM, inflammatory markers
Zickler et al^26^	Full text	Germany (2)	50 (47)	4 (+8 week extension study)	I: 58.1 ± 16.6 C: 59.8 ± 16.5	38	I: MCO-Ci400 C: Revaclear 400	Survival, adverse events, CRP, albumin (predialysis), MM, inflammatory markers
Non-randomized studies								
Cohort studies								
Cho et al^27^	Full text	Korea (1)	57 (57)	52	I: 53.7 ± 10.9 C: 56.4 ± 10.4	58	I: Theranova 400 C: FxCordiax 80	Survival, ERI, MM, cell-free hemoglobin
Yeter et al^28^	Full text	Turkey (1)	47 (42)	26	52.9 ± 16	63	I: Theranova 400 C: CorDiax 800	Survival, ERI, iron utilization, CRP, albumin (predialysis)
Before-after studies								
Alarcon et al^16^	Full text	Colombia (12)	992 (661)	52	60.5 ± 15.1	62	I: Theranova C: HF	QoL (KDQOL), dialysis symptoms index, restless legs syndrome
Albrizio et al^29^	Abstract	Italy (1)	8 (8)	2	78 ± 14	25	I: Theranova 400 C: HF	Adverse events, myoglobin
Ariza et al (Sanabria, 2020)^17^	Full text	Colombia (3)	81 (81)	104	61.1 ± 12.6	52	I: Theranova 400, 500 C: Polyflux 140, Revaclear 300, 400	Hospitalization, hospitalization days, ERI, iron utilization
Baharani, 2017a^30^	Abstract	England (1)	8 (8)	9	71 ± 11.8	75	I: Theranova 400 C: FxCordiax 60, 80	Adverse events, MM
Baharani et al^30^	Abstract	England (1)	18 (18)	8	73 ± 16.7	78	I: Theranova 400 C: Revaclear 300	Adverse events, MM
Bolton et al^31^	Full text (pre-print)	UK (1)	89 (58)	52	73 ± 12	61	I: Theranova C: Revaclear	Minutes to recover, symptoms (POS-S renal) and symptom severity
Bunch et al^a 18^	Full text	Colombia (1)	992 (638)	52	60 ± 15	62	I: Theranova C: HF	Survival, hospitalization, safety, albumin (predialysis)
D’Achiardi et al^32^	Abstract	Colombia (multiple)	52 (41)	24	61 ± 13	65	I: MCO C: HF	Adverse events, albumin (predialysis), MM inflammatory markers, CRP
García-Prieto et al^33^	Full text	Spain (1)	18 (18)	3	65 ± 13	50	I: Theranova 500 C: FxCordiax 80VR	Adverse events, albumin loss and RR, MM
Gernone et al^34^	Abstract	Italy (1)	11 (11)	52	70.8 ± 9	73	I: Theranova C: HF	PCS, MCS, ERI, albumin (predialysis), MM
Kim et al^35^	Full text	Korea (1)	6 (6)	3	66.1 ± 9.1	100	I: Theranova 400 C: Rexeed-21A	Adverse events, albumin loss, albumin RR, MM
Krishnasamy et al^36^	Full text	Australia and New Zealand (9)	89 (79)	32	66 ± 14	62	I: Theranova 400 C: Revaclear 400	Adverse events, QoL, RLS, ERI, CRP, albumin (predialysis)
Penny et al^37^	Abstract	Canada (2)	28 (23)	12	65.8 ± 14.3	52	I: Theranova C: HF	QoL (LEVIL)
Crossover studies								
Cozzolino et al^19^	Full text	Italy (1)	21 (20)	26	71 ± 13	76	I: Theranova 400 C: FX8, FX10, FX80, FX100, BK1.6, BG2.1	Survival, hospitalization, infection, albumin (predialysis), inflammatory markers

*Note.* QoL = quality of life; KDQOL = Kidney Disease Quality of Life Instrument; ERI = erythropoiesis resistance index; MM = middle-molecules (removal, reduction ratios, or predialysis serum levels); aPTT = activated partial thromboplastin time; EQ-5D-5L = EuroQol 5-Dimension Questionnaire; RR = risk ratio; MCO = medium cut-off membrane; CRP = C-reactive protein; HF = high-flux (not otherwise specified); NR = not reported; PMMA = polymethylmethacrylate; POS-S Renal = Palliative Care Outcome Scale–Symptom module for renal patients; PS = polysulfone.

a Single-arm MCO-HD data without comparator group for survival and hospitalization outcomes; not amenable to meta-analysis.

### Risk of Bias Within Studies

Risk of bias graphs are presented along with Forest plots in Online Appendix E, with detailed study-level risk of bias assessments in Online Appendix F. All studies were open label. For studies reporting PROs, we considered the risk of bias due to a lack of blinding as low risk of bias (see discussion). Factors contributing to risk of bias included attrition,^16-18,28,32,37^ lack of risk adjustment,^27^ and selection bias.^17^ We found no evidence of selective reporting or publication bias.

### Synthesis of Results

Effect estimates and certainty ratings for clinical outcomes are in the abbreviated GRADE Summary of Findings Table (Table 2; which presents only the estimate with the higher level of certainty for each outcome) with a complete table in Online Appendix G. Detailed explanations for certainty ratings are provided in the table footnotes.

**Table 2.** GRADE Summary of Findings Table (Explanations for Certainty Ratings in Table Footnotes).

Outcome No. of participants (studies)	Relative effect (95% CI)	Anticipated absolute effects* (95% CI)			Certainty	What happens
		With high-flux	With MCO-HD	Difference		
All-cause mortality assessed with: number of deaths from any cause Follow-up: range 12-52 weeks No. of participants: 306 (4 RS)	**RR 0.93** (0.31 to 2.78)	3.1%	**2.9%** (1 to 8.6)	**0.2% fewer** (2.1 fewer to 5.5 more)	⨁⨁◯◯ LOW^a^	MCO-HD may result in little to no difference in survival
All-cause mortality assessed with: number of deaths from any cause Follow-up: range 26-52 weeks No. of participants: 166 (4 NRS)	**RR 0.85** (0.12 to 5.91)	2.1%	**1.8%** (0.3 to 12.4)	**0.3% fewer** (1.9 fewer to 10.3 more)	⨁⨁◯◯ LOW^a^	MCO-HD may result in little to no difference in survival
All-cause mortality assessed with: number of deaths from any cause Follow-up: range 12-52 weeks No. of participants: 597 (4 RS and 4 NRS)	Not estimable	2.1% (4.4 events/100 person-years)	**1.6%** (3.3 events/100 person-years)	**0.4% fewer** (2.8 fewer to 2.1 more)	⨁⨁⨁◯ MODERATE^b^	MCO-HD likely results in little to no difference in survival
Hospitalization assessed with: number of episodes of hospitalization for any reason Follow-up: 24 weeks No. of participants: 172 (1 RS)	**Rate ratio 0.48** (0.27 to 0.84)	43.0%	**20.7%** (11.6 to 36.1)	**22.4% fewer** (31.4 fewer to 6.9 fewer)	⨁⨁◯◯ LOW^b,c^	MCO-HD may result in a reduction in hospitalization
Hospitalization length of stay assessed with: number of days in hospital Follow-up: 52 weeks No. of participants: 81 (1 NRS)	—	The mean hospitalization length of stay was **5.94** days	—	MD **1.5 days lower** (2.22 lower to 0.78 lower)	⨁⨁⨁◯ MODERATE^d^	MCO-HD likely reduces hospitalization days
Serious adverse events assessed with: death or any life-threatening condition leading to hospitalization Follow-up: range 12-26 weeks No. of participants: 312 (4 RS)	**Rate ratio 0.63** (0.38 to 1.04)	23.6%	**14.9%** (9 to 24.5)	**8.7% fewer** (14.6 fewer to 0.9 more)	⨁⨁◯◯ LOW^b,c^	MCO-HD may result in little to no difference in SAEs
Infection assessed with: number of infections requiring treatment Follow-up: range 24-26 weeks No. of participants: 113 (2 NRS)	**Rate ratio 0.38** (0.17 to 0.85)	17.2%	**6.5%** (2.9 to 14.6)	**10.6% fewer** (14.3 fewer to 2.6 fewer)	⨁⨁⨁◯ MODERATE^b,e^	MCO-HD likely reduces infection
Quality of life assessed with: London Evaluation of Illness (LEVIL); a higher score is better Follow-up: 12 weeks No. of participants: 28 (1 NRS)	—	The mean quality of life was **51** points	—	MD **16.67 points higher** (6.92 higher to 26.42 higher)	⨁⨁⨁◯ MODERATE^c,f^	MCO-HD likely results in a large increase in quality of life among individuals with low (<70/100) baseline scores
Burden of kidney disease assessed with: KDQOL; a higher score is better Follow-up: 52 weeks No. of participants: 992 (1 NRS)	—	The mean burden of kidney disease was **46** points	—	MD **4 points higher** (1.06 higher to 6.94 higher)	⨁⨁⨁◯ MODERATE^h^	MCO-HD likely results in a large increase in burden of kidney disease scores
Effects of kidney disease assessed with: KDQOL; a higher score is better Follow-up: 52 weeks No. of participants: 992 (1 NRS)	—	The mean effects of kidney disease was **70** points	—	MD **5.4 points higher** (3.23 higher to 7.57 higher)	⨁⨁⨁◯ MODERATE^h^	MCO-HD likely results in a large increase in effects of kidney disease scores
Symptoms/problem list assessed with: KDQOL; a higher score is better Follow-up: range 12-24 weeks No. of participants: 222 (2 RS)	—	The mean symptoms/problem list ranged from **70 to 81**	—	MD **0.14 higher** (2.79 lower to 3.08 higher)	⨁⨁◯◯ LOW^c,i^	MCO-HD may result in little to no difference in symptoms/problem list scores
Symptoms/problem list assessed with: KDQOL and E-SAS; a higher score is better Follow-up: range 24-52 weeks No. of participants: 1081 (2 NRS)	—	The mean symptoms/problem list ranged from **79 to 89**	—	MD **0.61 higher** (0.38 lower to 1.6 higher)	⨁⨁◯◯ LOW^c,j^	MCO-HD may result in little to no difference in kDQOL—Symptoms/Problem List
Pain assessed with: KDQOL; a higher score is better Follow-up: 12 weeks No. of participants: 50 (1 RS)	—	The mean pain was **69.3** points	—	MD **3 points lower** (12.05 lower to 6.05 higher)	⨁⨁◯◯ LOW^k^	MCO-HD may result in little to no difference in pain scores
Physical health assessed with: KDQOL—Physical Component Summary; a higher score is better Follow-up: range 12-24 weeks No. of participants: 222 (2 RS)	—	The mean physical health ranged from **44 to 52**	—	MD **0.75 higher** (1.35 lower to 2.86 higher)	⨁⨁◯◯ LOW^c,i^	MCO-HD may result in little to no difference in physical health
Physical health assessed with: KDQOL—Physical Component Summary; a higher score is better Follow-up: range 24-52 weeks No. of participants: 1003 (2 NRS)	—	The mean physical health ranged from **27 to 41**	—	MD **6.12 higher** (5.87 lower to 18.12 higher)	⨁⨁◯◯ LOW^c,l^	MCO-HD may result in little to no difference in physical health
Mental health assessed with: KDQOL—Mental Composite Summary; a higher score is better No. of participants: 1003 (2 NRS)	—	The mean mental health ranged from **44 to 51**	—	MD **4.61 higher** (3.01 lower to 12.24 higher)	⨁⨁⨁◯ MODERATE^c^	MCO-HD likely results in little to no difference in mental health
Pruritus assessed with: multidimensional pruritus questionnaire; a lower score is better Follow-up: 12 weeks No. of participants: 50 (1 RS)	—	The mean pruritus was **9.92**	—	MD **4.38 lower** (7.1 lower to 1.66 lower)	⨁⨁⨁◯ MODERATE^m^	MCO-HD likely reduces pruritus
Recovery time assessed with: minutes to recover questionnaire; intention-to-treat analysis; a lower score is better Follow-up: 52 weeks No. of participants: 89 (1 NRS)	—	The mean recovery time was **600** minutes	—	MD **420 Minutes lower** (541 lower to 299 lower)	⨁⨁⨁⨁ HIGH^n^	MCO-HD results in large reduction in minutes to recover after dialysis
Restless Legs Syndrome—NRS assessed with: NIH Diagnostic Criteria No. of participants: 992 (1 NRS)	**OR 0.39** (0.29 to 0.53)	22.1%	**10.0%** (7.6 to 13.1)	**12.2% fewer** (14.5 fewer to 9 fewer)	⨁⨁⨁◯ MODERATE^c^	MCO-HD likely results in a large reduction in restless legs syndrome
Symptom Severity—NRS assessed with: Palliative Care Outcome Scale—Symptom Module (Proportion of patients with 1 or more symptoms rated “severe” or “overwhelming”) Follow-up: 52 weeks No. of participants: 89 (1 NRS)	**OR 0.81** (0.76 to 0.86)	66.1%	**61.2%** (59.7 to 62.6)	**4.9% fewer** (6.4 fewer to 3.5 fewer)	⨁⨁⨁◯ MODERATE^m,o^	MCO-HD likely reduces symptom severity
Erythropoiesis resistance index Follow-up: 12 weeks No. of participants: 90 (2 RS)	—	The mean erythropoiesis resistance index was **15** U/kg/week/g/L achieved Hb	—	**2.92 U/kg/week/g/L acheived Hb lower** (4.25 lower to 1.6 lower)	⨁⨁⨁◯ MODERATE^b^	MCO-HD likely reduces erythropoiesis resistance index
Iron utilization assessed with: cumulative intravenous dose in 12 weeks (mg) Follow-up: 12 weeks No. of participants: 90 (2 RS)	—	The mean iron utilization ranged from **700 to 1000** mg	—	MD **293 mg lower** (368 lower to 218 lower)	⨁⨁⨁◯ MODERATE^b^	MCO-HD likely reduces iron utilization

*Note.* **GRADE Working Group grades of evidence**

**High certainty:** We are very confident that the true effect lies close to that of the estimate of the effect.

**Moderate certainty:** We are moderately confident in the effect estimate: The true effect is likely to be close to the estimate of the effect, but there is a possibility that it is substantially different.

**Low certainty:** Our confidence in the effect estimate is limited: The true effect may be substantially different from the estimate of the effect.

**Very low certainty:** We have very little confidence in the effect estimate: The true effect is likely to be substantially different from the estimate of effect.

CI = confidence interval; MCO-HD = medium cut-off hemodialysis; RS = randomized study; RR = risk ratio; NRS = nonrandomized study; MD = mean difference; SAE = serious adverse events; KDQOL = kidney disease quality of life instrument; E-SAS = Edmonton Symptom Assessment System; NIH = National Institutes of Health; OR = odds ratio; RoB = risk of bias.

Explanations

a Total event count <10 across study populations, does not meet optimal information size criterion.

b Small overall sample size; optimal information size criterion not met.

c A high rate of attrition may have introduced serious risk of bias.

d Arizia 2021 (N = 81) was the larger among the studies reporting hospitalization outcomes and used a before-after design in which patients were enrolled only if they had a full year of follow-up before and after switching to MCO-HD. This design is high RoB due to selection and survivor bias, regression to the mean, and maturation/effect of time.

e Effect estimate is based on 2 NRS with before-after design and excludes 1 NRS (cohort design) which is higher RoB due to case-mix differences without statistical adjustment.

f Estimate is based on a subgroup of patients with low baseline QoL scores and results may not be applicable to patients with normal or high baseline scores.

g QoL scores increased in a linear fashion over time during treatment with MCO-HD, then decreased linearly after crossing over to high-flux membranes; consistent with a dose-response effect; however, since the study has potential bias due to attrition, we did not rate up for dose-response or large effects.

h The high rate of attrition introduces risk of survivor bias on patient-reported outcome measures in the large observational study.

i In rating the certainty that there is a treatment effect (threshold: null effect), the confidence interval crosses this threshold; OIS criterion not met.

j *I*^2^ = 93%. In the study by Krishnasamy 2020, patients in the control group had scores of 89/100, resulting in a ceiling effect, contributing to inconsistency across studies.

k Confidence interval includes appreciable benefit and harm (using 5 scalar units as the minimal important difference for KDQOL subscales); downgraded 2 levels for imprecision.

l *I*^2^ = 89%; confidence intervals do not overlap.

m Small overall sample size in a single study; optimal information size criterion not met.

n Although the study was prone to survivor bias due to attrition, a per-protocol analysis confirmed the magnitude and direction of effect observed in the ITT analysis; hence, we did not rate down for RoB.

o Although this single study had significant attrition, this outcome was based on a per-protocol analysis that we considered low risk of bias, and we therefore did not rate down.

* **The absolute risk** in the MCO-HD group (and its 95% confidence interval) is calculated based on the assumed risk (which is equal to the mean risk across studies included in the estimate) in the high-flux group and the **relative effect (and** its 95% CI).

### Major Clinical Events

#### All-cause mortality

Four randomized and 4 nonrandomized studies with 136.7 and 152.0 patient-years had zero events in 11 out of 16 arms. Imputing a continuity correction of 0.5 for zero cells found that MCO dialysis may have little to no effect on mortality, but with low certainty, downgraded 2 levels for imprecision. To avoid bias from imputation, we calculated the risk difference (RD) by pooling events across randomized and nonrandomized studies and found with moderate certainty that MCO dialysis likely has little to no effect on mortality (RD = −0.4%, 95% confidence interval [CI], −2.8 to 2.1). One large single-arm study measured crude mortality at 8.5 deaths per 100 person-years (95% CI, 6.8 to 10.7) in a cohort of 992 patients with 866 person-years of follow-up^18^ (very low certainty for comparative effect).

#### Hospitalization for any cause

One randomized study with 78.7 person-years provided low certainty evidence that MCO dialysis may result in a reduction in hospitalization with a rate ratio of 0.48 (95% CI, 0.27 to 0.84), downgraded for risk of bias and imprecision. Two nonrandomized studies with 221.1 combined patient-years showed similar effects but with very low certainty.

#### Hospitalization length of stay

One nonrandomized study with 162 patient-years found that MCO dialysis likely reduced mean length of stay by a mean difference of −1.5 days (95% CI, −2.22 to −0.78); moderate certainty, downgraded for risk of bias.

#### Serious adverse events

Four trials that reported serious adverse events (SAEs; defined as fatal or life-threatening events leading to hospitalization) provided low certainty of little to no difference with a rate ratio of 0.63 (95% CI, 0.38 to 1.04; *I*^2^ = 3%) with comparable relative risk. Seven nonrandomized studies^18,27,29,32,33,35,36^ (not pooled due to lack of standard outcome definitions; very low certainty due to lack of comparative data) explicitly stated that there were no dialysis-related complications attributable to MCO dialysis.

#### Infection

Two nonrandomized studies with 68.8 patient-years found that that MCO dialysis likely reduces infection with a rate ratio of 0.38 (95% CI, 0.17 to 0.85; *I*^2^ = 0%) with similar effects using a relative risk; moderate certainty, downgraded for imprecision.

### Patient-Reported Outcomes

#### Quality of life

Two randomized studies found little to no difference in overall quality of life with MCO dialysis, with low certainty due to risk of bias and imprecision. One small nonrandomized study reported an improvement of 16.7/100 points on a novel instrument (the London Evaluation of Illness [LEVIL] questionnaire) in a subgroup of patients who had baseline scores <70/100. We downgraded one level for risk of bias due to 18% attrition during the 3-month study. Scores increased in a linear fashion over consecutive dialysis sessions and then returned to baseline after an 8-week wash out period, consistent with a dose-response effect. As the estimate was potentially biased, we did not rate up for large effect size or dose-response.

#### Burden of kidney disease

Two randomized studies with 150 participants found little to no difference in the KDQOL Burden subscale; low certainty (risk of bias and imprecision). One large nonrandomized study with 993 subjects followed for a year reported an improvement of 4.0 points (95% CI, 1.1 to 6.9) with MCO dialysis, with moderate certainty downgraded one level for risk of bias.

#### Effects of kidney disease

Two randomized studies found little to no difference on KDQOL Effects (low certainty). Scores in the high-flux group were between 68 and 77 points, potentially creating a ceiling effect. A nonrandomized study with 993 subjects demonstrated an improvement of 5.4 points (95% CI, 3.2 to 7.6) after 1 year of treatment with MCO dialysis, with moderate certainty, downgraded one level for risk of bias.

#### Symptoms/problem list

Both randomized and nonrandomized studies provided low certainty of little to no difference in the KDQOL symptoms subscale. Both bodies of evidence were downgraded one level for risk of bias, and one additional level for imprecision (randomized studies), and inconsistency (nonrandomized studies). As with the KDQOL Effects subscale, mean scores in the comparator group were 70 to 81 (randomized studies) and 79 to 89 (nonrandomized studies), potentially creating a ceiling effect for this outcome.

#### Pain

One randomized study with 49 subjects provided low certainty of little to no difference (MD = −3.0; 95% CI, −12.05 to 6.05) downgraded 2 levels for imprecision since the confidence interval included both appreciable benefit and harm using a minimal important difference (MID) threshold of 5 scale units.

#### Physical health

Pooled estimates from randomized and nonrandomized studies provided low certainty of little to no difference with certainty downgraded for risk of bias in both bodies of evidence, and one additional level for imprecision (randomized studies), and inconsistency (nonrandomized studies). Excluding one nonrandomized study^34^ published as an abstract provided similar results.

#### Mental health

The pooled estimate from 2 randomized studies provided low certainty of little to no difference, downgraded for risk of bias and imprecision. The pooled estimate from nonrandomized studies provided moderate certainty of no effect with the upper, but not the lower bound exceeding the 5-point MID threshold. Excluding one nonrandomized study^34^ published as an abstract provided similar results.

#### Pruritus

A single randomized study with 49 participants found that MCO dialysis likely reduces pruritus with MD −4.4 points on a 45-point scale (95% CI, −7.1 to −1.66), with moderate certainty, downgraded for imprecision. Using a 10-point visual analog scale, the same study found a reduction in pruritus scores of −1.18 (95% CI, −2.05 to −0.31).

#### Symptom severity

One nonrandomized study measured the proportion of patients with 1 or more symptom rated as “severe” or “overwhelming” at baseline and 1 year. The odds ratio for a reduction in symptom severity with MCO dialysis was 0.81 (95% CI, 0.76 to 0.86); moderate certainty, downgraded for imprecision.

#### Recovery time

One nonrandomized study found with high certainty that a year of treatment with MCO dialysis reduced recovery time by −420 minutes (95% CI, −540 to −299), using a validated instrument.^39^ Although the study had potential risk of bias due to patient attrition, a per-protocol analysis found similar results, so we did not downgrade.

#### Restless legs syndrome

One large nonrandomized study measured a reduction in the prevalence of restless legs syndrome (based on NIH diagnostic criteria)^40^ from 22.1% at baseline to 10.0%, 1 year after converting to MCO dialysis with odds ratio 0.39 (95% CI, 0.29 to 0.53; moderate certainty downgraded for risk of bias due to attrition).

### Other Safety Outcomes

#### Dialyzer reactions

One study of 130 601 hemodialysis sessions reported no Type A or Type B dialyzer reactions with MCO dialysis.^18^

### Medication Utilization

#### Erythropoiesis resistance index

The pooled mean difference for erythropoiesis resistance index (ERI) was −2.92 U/kg/week/g/L achieved hemoglobin (95% CI, −4.25 to −1.6; *I*^2^ = 0%) with MCO dialysis, in 2 randomized studies with moderate certainty, downgraded for imprecision. With mean ERI 13-15 U/kg/week/g/L in the high-flux arms, this represents a 20% to 23% reduction in erythropoiesis stimulating agent (ESA) use. One randomized study found a linear decrease in ERI over time with MCO dialysis, supporting a true causal effect. Results were similar in nonrandomized studies, including a subgroup of 3 studies with 1 year of follow-up, but with low certainty.

#### Iron utilization

The pooled mean difference in cumulative intravenous iron use over 12 weeks was −293 mg (95% CI, −368 to −218; *I*^2^ = 93%), favoring MCO dialysis, downgraded for imprecision (contributing to inconsistency). With iron use between 700 and 1000 mg in the high-flux groups, this represents 29% to 42% less iron use with MCO dialysis. Results were similar in nonrandomized studies, but with low certainty.

## Discussion

### Principal Findings

This meta-analysis provides high certainty evidence that compared with high-flux membranes, MCO dialysis reduces recovery time after hemodialysis. We found with moderate certainty that MCO dialysis likely reduces infection, hospital length of stay, overall quality of life, KDQOL burden and effects of kidney disease, pruritus, restless legs syndrome, symptom severity, ERI, and iron utilization. We further found with low certainty that MCO dialysis may result in little to no effect on mortality and SAEs but may result in a reduction in hospitalization rates. We found with low certainty that MCO dialysis had little to no effect on KDQOL symptoms/problem list, pain, and physical health and moderate certainty that MCO dialysis likely has no effect on the KDQOL mental health composite.

### Strengths and Limitations of This Review

Strengths of this review include adherence to a registered protocol, a sensitive search strategy, independent screening, data extraction, and quality appraisal in duplicate. We used GRADE in all aspects of the review and used rigorous risk of bias assessment tools. Three members of our team with extensive experience with GRADE methods independently assessed the certainty of evidence. We guarded against bias in the meta-analysis of PRO measures and domains by enlisting a blinded collaborator to create appropriate groupings for meta-analysis.

### Comparisons With Previous Research

To our knowledge, this is the first systematic review of MCO dialysis, which we report in 2 parts. In the second accompanying report of laboratory-based surrogate outcomes,^41^ we found that MCO dialysis provided greater clearance and reduced predialysis concentrations of representative solutes including β-2-microglobulin, κ- and λ-light chains and myoglobin, and reduced mRNA expression of interleukin (IL)-6 and tumor necrosis factor (TNF)-α in peripheral leukocytes. These and other solutes of comparable molecular weight have been associated with uremic symptoms, impaired immunity, cardiovascular disease, and other adverse effects. Thus, our findings of improved PROs, infection rates, and lower erythropoietin and iron requirements are congruent with the underlying physiological effects of the MCO membrane.

To date, studies of MCO dialysis have largely focused on biomarkers and PROs, with no studies powered for survival or hospitalization events, leaving some important evidence gaps. In this meta-analysis, the crude mortality rate in the control group of 4.4 deaths per 100 person-years is consistent with previous hemodialysis trials,^42^ but several-fold lower compared with the general hemodialysis population.^43^ While this provides some reassurance of safety, it also highlights a major challenge in comparative effectiveness dialysis trials, which is the over-representation of low-risk, healthy individuals.^44^ Given the sparsity of directly comparative data, it is worth considering insights from large single-arm studies. A Colombian registry with 992 participants with 866 person-years measured 8.5 deaths/100 person-years (95% CI, 6.8 to 10.7) with MCO dialysis, while the same provider reported 14.6 deaths/100 patient-years when high-flux membranes were in use.^45^ In the United States, crude mortality rates are higher still at 15 to 29 deaths/100 person-years with high-flux membranes.^46^ Collectively, these data suggest no obvious excess mortality with MCO dialysis, but confirmatory trials are needed. Such studies could provide additional information on other SAEs, which also appear to be rare.

A single randomized study found that MCO dialysis may reduce hospitalization for any cause (low certainty) but did not report cause of hospitalization. One nonrandomized study reported lower hospitalization rates and length of stay, largely driven by reduced infection rates.^17^ This is consistent with our pooled rate ratio for infection (0.38; 95% CI, 0.17 to 0.85), which provides moderate certainty, downgraded for imprecision, again due to a limited number of events across studies. The notion that enhanced large middle-molecule clearance could reduce infection rates has motivated large trials of convective therapies, though the largest of these found no significant effect with hemodiafiltration.^47^ Although we did not directly compare MCO dialysis with convective therapies, it is plausible that differences in membrane characteristics, substitution fluid volumes, and other treatment parameters could result in different depuration profiles that could lead to differences in outcomes; hence, studies directly comparing these modalities are likely to be of interest, and some are underway.^48,49^

While quality of life generally declines over time on maintenance hemodialysis,^50^ MCO dialysis improved several well-validated PRO measures.^39,40,51,52^ The MID is the minimum change in a score that is perceptible and important to patients, and is between 2.5 and 5.0 scale units for SF-36/KDQOL subscales,^53^ with a threshold of 4.0 used in sample size determinations for previous hemodialysis trials.^54^ Treatment with MCO dialysis exceeded and met the MID thresholds for the Effects (5.4) and Burden (4.0) subscales with moderate certainty. Such effects are important not only as direct measures of quality of life but also for their prognostic importance given their strong associations with survival and hospitalization.^55^ The study by Penny et al^37^ used a novel quality of life instrument (LEVIL) administered at consecutive dialysis sessions with a large effect in patients with baseline scores below 70/100, highlighting the utility of separating potential responders from non-responders who might otherwise exhibit ceiling effects. The presence of ceiling effects might explain the apparent lack of effect with MCO dialysis on the KDQOL Symptoms domain for which baseline scores ranged between 70 and 90 across studies. MCO dialysis also improved recovery time, symptom severity, and the prevalence of restless legs syndrome. Recovery time also associates with mortality and hospitalization^56^ and is improved with frequent hemodialysis,^57^ but not with conventional hemodialysis,^58^ suggesting a causal role for enhanced large middle-molecule clearance with therapies that can achieve it.

Finally, MCO dialysis likely reduced erythropoietin resistance and iron requirements in medium-term (12-week) randomized studies (moderate certainty), with qualitatively similar effects in nonrandomized studies with long-term (1 year) follow-up (low certainty). Although it is beyond the scope of this review to elucidate the molecular mechanisms underlying these effects, it is worth noting that enhanced clearance of hepcidin (a middle molecule) and inflammatory mediators could be implicated,^59^ as has been reported with convective therapies.^60^

### Certainty of the Evidence

We recognize several important limitations in this body of evidence. Most outcomes were based on a small number of studies, many of which were nonrandomized. Studies were relatively small and major clinical events were rare, resulting in downgrading for imprecision. As with other hemodialysis trials, study withdrawal was high, especially in long-term trials. Where reported, reasons for withdrawal were similar between groups and were thus non-differential. Nevertheless, we downgraded most estimates for risk of bias where studies with high rates of attrition carried significant weight. All included studies were open label, which is typical in dialysis trials. However, for several reasons, we did not rate down further for open-label design. As with previous dialysis trials, we expected that patients’ limited recall of prior scores as well as waning enthusiasm (for receiving a novel therapy) over a long-term study should have mitigated any intentional or unintentional bias in PRO scores.^57^ Meta-analyses comparing treatment effects in open-label and blinded studies in other chronic disease populations support this reasoning.^61^ Moreover, one study found a linear increase in quality of life scores over time on MCO dialysis, with a return to baseline after washout, supporting a potential causal effect rather than satisficing or manipulating of scores.^37^ Importantly, as all randomized and nonrandomized studies in this review were open label, downgrading one additional level for this factor would not have helped us to differentiate levels of certainty between these bodies of evidence. Nevertheless, users of this review can at their discretion rate down an additional level if it aids their decision-making. Finally, industry-sponsored studies are potentially at risk for publication bias. As all relevant trials registered at clinicaltrials.gov were either reported and included in this review, or ongoing (N=2), and given the available funnel plots for selected outcomes, we did not consider the risk of publication bias serious enough to warrant rating down.

Despite these limitations, there were several factors that increased our overall confidence in the estimates of effect. The consistency and concordance of the observed treatment effects, that is, improvements across most PROs, and the concordance of these effects with the changes in relevant biomarkers also increase our certainty in the evidence. Moreover, all but 4 studies (2 parallel-arm randomized studies and 2 cohort studies)^20-22,27,28^ were before-after designs or crossover trials, in which patients served as their own controls. Since these studies were able to exploit paired analysis designs, they provided much higher statistical power than would have been possible with unpaired analyses. As a result, estimates derived from seemingly small numbers of studies with small study populations met the optimal information size criterion and did not warrant downgrading for imprecision. Finally, given the nature of the intervention, the only potential carryover effect that we anticipated in crossover studies was a sustained reduction in large middle-molecule concentrations after switching to high-flux membranes. Such an effect would have biased all outcome measures toward the null, further increasing our certainty in the evidence.

### Implications for Decision-Making

This review of 22 studies including 6 randomized trials provides detailed information for consideration by decision-makers on benefits and harms of a novel dialysis membrane with enhanced LMM clearance. In performing this review, we appraised certainty for each outcome on an individual basis and did not adjudicate the overall certainty across outcomes as might be done in a practice guideline or coverage decision. Decision-makers applying our findings using GRADE would prioritize outcomes and determine the overall certainty across those deemed critical in their specific contexts. Contextualization of effect sizes and related imprecision judgments, values and preferences, implementation issues, and costs would require further value judgments that are likely to vary across populations, health systems, and payors. It is noteworthy that compared with intensive hemodialysis and convective therapies, substituting MCO for other membranes is straightforward and does not require additional training or equipment.

Users of this review are also likely to consider its applicability to their target populations. Given the physiology underlying its effects, it seems likely that MCO dialysis should produce similar outcomes across populations and practice settings. Generalizability is further supported by the diversity of the populations represented in the included studies. Although outcomes improved in the overall study populations, patients with low baseline health status or high symptom burden might reap the greatest benefits from MCO dialysis, and greater absolute effects might be achieved in populations with higher baseline risk for outcomes such as infection.

## Conclusions

The MCO dialyzer improved a range of outcomes with concordant signals of benefit, and in a manner consistent with its anticipated mechanism of effect. While the current available evidence for MCO dialysis is of predominantly moderate certainty, promising innovations in dialysis care are scarce and thus likely to generate interest as the evidence base evolves. The notion that patient-important outcomes can be improved by simply substituting a dialysis membrane is appealing and could by virtue of its scalability, impact patient care, and by its novelty stimulate further innovation. Although larger studies would be needed to further quantify any effects of MCO dialysis on major clinical events, to date, there are no signals in the published literature to suggest risk or harm with this device. Given the very low event rates in trials to date, future studies powered for mortality and other major outcomes could be impracticably large; hence, alternate designs such as registry-based cluster randomized trials, prospective cohort studies, and ongoing surveillance might help fill these evidence gaps.

## Supplemental Material

sj-docx-1-cjk-10.1177_20543581211067087 – Supplemental material for Clinical Outcomes With Medium Cut-Off Versus High-Flux Hemodialysis Membranes: A Systematic Review and Meta-Analysis Click here for additional data file. Supplemental material, sj-docx-1-cjk-10.1177_20543581211067087 for Clinical Outcomes With Medium Cut-Off Versus High-Flux Hemodialysis Membranes: A Systematic Review and Meta-Analysis by Maryam Kandi, Romina Brignardello-Petersen, Rachel Couban, Celina Wu and Gihad Nesrallah in Canadian Journal of Kidney Health and Disease

sj-docx-2-cjk-10.1177_20543581211067087 – Supplemental material for Clinical Outcomes With Medium Cut-Off Versus High-Flux Hemodialysis Membranes: A Systematic Review and Meta-Analysis Click here for additional data file. Supplemental material, sj-docx-2-cjk-10.1177_20543581211067087 for Clinical Outcomes With Medium Cut-Off Versus High-Flux Hemodialysis Membranes: A Systematic Review and Meta-Analysis by Maryam Kandi, Romina Brignardello-Petersen, Rachel Couban, Celina Wu and Gihad Nesrallah in Canadian Journal of Kidney Health and Disease

sj-docx-3-cjk-10.1177_20543581211067087 – Supplemental material for Clinical Outcomes With Medium Cut-Off Versus High-Flux Hemodialysis Membranes: A Systematic Review and Meta-Analysis Click here for additional data file. Supplemental material, sj-docx-3-cjk-10.1177_20543581211067087 for Clinical Outcomes With Medium Cut-Off Versus High-Flux Hemodialysis Membranes: A Systematic Review and Meta-Analysis by Maryam Kandi, Romina Brignardello-Petersen, Rachel Couban, Celina Wu and Gihad Nesrallah in Canadian Journal of Kidney Health and Disease

sj-docx-4-cjk-10.1177_20543581211067087 – Supplemental material for Clinical Outcomes With Medium Cut-Off Versus High-Flux Hemodialysis Membranes: A Systematic Review and Meta-Analysis Click here for additional data file. Supplemental material, sj-docx-4-cjk-10.1177_20543581211067087 for Clinical Outcomes With Medium Cut-Off Versus High-Flux Hemodialysis Membranes: A Systematic Review and Meta-Analysis by Maryam Kandi, Romina Brignardello-Petersen, Rachel Couban, Celina Wu and Gihad Nesrallah in Canadian Journal of Kidney Health and Disease

sj-pptx-5-cjk-10.1177_20543581211067087 – Supplemental material for Clinical Outcomes With Medium Cut-Off Versus High-Flux Hemodialysis Membranes: A Systematic Review and Meta-Analysis Click here for additional data file. Supplemental material, sj-pptx-5-cjk-10.1177_20543581211067087 for Clinical Outcomes With Medium Cut-Off Versus High-Flux Hemodialysis Membranes: A Systematic Review and Meta-Analysis by Maryam Kandi, Romina Brignardello-Petersen, Rachel Couban, Celina Wu and Gihad Nesrallah in Canadian Journal of Kidney Health and Disease

sj-xlsx-6-cjk-10.1177_20543581211067087 – Supplemental material for Clinical Outcomes With Medium Cut-Off Versus High-Flux Hemodialysis Membranes: A Systematic Review and Meta-Analysis Click here for additional data file. Supplemental material, sj-xlsx-6-cjk-10.1177_20543581211067087 for Clinical Outcomes With Medium Cut-Off Versus High-Flux Hemodialysis Membranes: A Systematic Review and Meta-Analysis by Maryam Kandi, Romina Brignardello-Petersen, Rachel Couban, Celina Wu and Gihad Nesrallah in Canadian Journal of Kidney Health and Disease

sj-docx-7-cjk-10.1177_20543581211067087 – Supplemental material for Clinical Outcomes With Medium Cut-Off Versus High-Flux Hemodialysis Membranes: A Systematic Review and Meta-Analysis Click here for additional data file. Supplemental material, sj-docx-7-cjk-10.1177_20543581211067087 for Clinical Outcomes With Medium Cut-Off Versus High-Flux Hemodialysis Membranes: A Systematic Review and Meta-Analysis by Maryam Kandi, Romina Brignardello-Petersen, Rachel Couban, Celina Wu and Gihad Nesrallah in Canadian Journal of Kidney Health and Disease

## References

[1] Ronco C La Manna G. Expanded hemodialysis: a new therapy for a new class of membranes. Contrib Nephrol. 2017;190:124-133. doi:10.1159/000468959. 28535525

[2] Sterne JAC Savovic J Page MJ , et al. RoB 2: a revised tool for assessing risk of bias in randomised trials. BMJ. 2019;366:l489820190830. doi:10.1136/bmj.l4898. 31462531

[3] JPTH, Tianjing L Sterne J . Revised Cochrane risk of bias tool for randomized trials (RoB 2) Additional considerations for crossover trials. https://www.riskofbias.info/welcome/rob-2-0-tool/rob-2-for-crossover-trials. Published 2021. Accessed April 1, 2021.

[4] Sterne JA Hernán MA Reeves BC , et al. ROBINS-I: a tool for assessing risk of bias in non-randomised studies of interventions. BMJ. 2016; i4919. doi:10.1136/bmj.i4919. PMC5062054 27733354

[5] Johnston BC Patrick DL Busse JW , et al. Patient-reported outcomes in meta-analyses–Part 1: assessing risk of bias and combining outcomes. Health Qual Life Outcomes. 2013;11:10920130703. doi:10.1186/1477-7525-11-109. PMC3708764 23815754

[6] Guyatt GH Oxman AD Santesso N , et al. GRADE guidelines: 12. Preparing summary of findings tables-binary outcomes. J Clin Epidemiol. 2013;66:158-172. doi:10.1016/j.jclinepi.2012.01.012. 22609141

[7] Guyatt GH Oxman AD Vist G , et al. GRADE guidelines: 4. Rating the Quality of evidence–study limitations (risk of bias). J Clin Epidemiol. 2011;64:407-415. doi:10.1016/j.jclinepi.2010.07.017. 21247734

[8] Schunemann HJ Cuello C Akl EA , et al. GRADE guidelines: 18. How ROBINS-I and other tools to assess risk of bias in nonrandomized studies should be used to rate the certainty of a body of evidence. J Clin Epidemiol. 2019;111:105-114. doi:10.1016/j.jclinepi.2018.01.012. 29432858 PMC6692166

[9] Guyatt GH Oxman AD Kunz R , et al. GRADE guidelines: 7. Rating the quality of Evidence–inconsistency. J Clin Epidemiol. 2011;64:1294-1302. doi:10.1016/j.jclinepi.2011.03.017. 21803546

[10] Guyatt GH Oxman AD Kunz R , et al. GRADE guidelines: 8. Rating the quality of evidence–indirectness. J Clin Epidemiol. 2011;64:1303-1310. doi:10.1016/j.jclinepi.2011.04.014. 21802903

[11] Guyatt GH Oxman AD Kunz R , et al. GRADE guidelines 6. Rating the quality of evidence–imprecision. J Clin Epidemiol. 2011;64:1283-1293. doi:10.1016/j.jclinepi.2011.01.012. 21839614

[12] Guyatt GH Oxman AD Montori V , et al. GRADE guidelines: 5. Rating the quality of evidence–publication bias. J Clin Epidemiol. 2011;64:1277-1282. doi:10.1016/j.jclinepi.2011.01.011. 21802904

[13] Guyatt GH Oxman AD Sultan S , et al. GRADE guidelines: 9. Rating up the quality of evidence. J Clin Epidemiol. 2011;64:1311-1316. doi:10.1016/j.jclinepi.2011.06.004. 21802902

[14] Newcombe RG Bender R. Implementing GRADE: calculating the risk difference from the baseline risk and the relative risk. Evid Based Med. 2014;19(1):6-8. doi:10.1136/eb-2013-101340. 23970740 PMC3913115

[15] Santesso N Glenton C Dahm P , et al. GRADE guidelines 26: informative statements to communicate the findings of systematic reviews of interventions. J Clin Epidemiol. 2019;119:126-135. doi:10.1016/j.jclinepi.2019.10.014. 31711912

[16] Alarcon JC Bunch A Ardila F , et al. Impact of medium cut-off dialyzers on patient-reported outcomes: COREXH registry. Blood Purif. 2021;50(1):110-118. doi:10.1159/000508803. 33176299

[bibr17-20543581211067087] Ariza JG Walton SM Suarez AM Sanabria M Vesga JI. An initial evaluation of expanded hemodialysis on hospitalizations, drug utilization, costs, and patient utility in Colombia. Ther Apher Dial. 2021;25(5):621-627. doi:10.1111/1744-9987.13620. 33403817 PMC8451823

[bibr18-20543581211067087] Bunch A Sanchez R Nilsson LG , et al. Medium cut-off dialyzers in a large population of hemodialysis patients in Colombia: COREXH registry. Ther Apher Dial. 2021;25(1):33-43. doi:10.1111/1744-9987.13506. 32352233 PMC7818220

[bibr19-20543581211067087] Cozzolino M Magagnoli L Ciceri P Conte F Galassi A. Effects of a medium cut-off (Theranova®) dialyser on haemodialysis patients: a prospective, cross-over study. Clin Kidney J. 2021;14:382-389. doi:10.1093/ckj/sfz155. 33564442 PMC7857781

[bibr20-20543581211067087] Lim J-H Jeon Y Yook J-M , et al. Medium cut-off dialyzer improves erythropoiesis stimulating agent resistance in a hepcidin-independent manner in maintenance hemodialysis patients: results from a randomized controlled trial. Sci. 2020;10:16062. doi:10.1038/s41598-020-73124-x. PMC7524751 32994531

[bibr21-20543581211067087] Weiner DE Falzon L Skoufos L , et al. Efficacy and safety of expanded hemodialysis with the theranova 400 dialyzer: a randomized controlled trial. Clin J Am Soc Nephrol. 2020;15:1310-1319. doi:10.2215/cjn.01210120. 32843372 PMC7480550

[bibr22-20543581211067087] Lim JH Park Y Yook JM , et al. Randomized controlled trial of medium cut-off versus high-flux dialyzers on quality of life outcomes in maintenance hemodialysis patients. Sci. 2020;10:7780. doi:10.1038/s41598-020-64622-z. PMC7210312 32385307

[bibr23-20543581211067087] Belmouaz M Bauwens M Hauet T , et al. Comparison of the removal of uraemic toxins with medium cut-off and high-flux dialysers: a randomized clinical trial. Nephrol Dial Transplant. 2020;35:328-335. doi:10.1093/ndt/gfz189. 31578564

[bibr24-20543581211067087] Santos A Macías N Vega A , et al. Efficacy of enoxaparin in preventing coagulation during high-flux haemodialysis, expanded haemodialysis and haemodiafiltration. Clin Kidney J. 2021;14(4):1120-1125. doi:10.1093/ckj/sfaa057. 33841857 PMC8023216

[bibr25-20543581211067087] Sevinc M Hasbal NB Yilmaz V , et al. Comparison of circulating levels of uremic toxins in hemodialysis patients treated with medium cut-off membranes and high-flux membranes: theranova in Sisli Hamidiye Etfal (THE SHE) Randomized Control Study. Blood Purif. 2020;49(6):733-742. doi:10.1159/000508061. 32634815

[26] Zickler D Schindler R Willy K , et al. Medium Cut-Off (MCO) membranes reduce inflammation in chronic dialysis patients-a randomized controlled clinical trial. PLoS ONE. 2017;12:e0169024. doi:10.1371/journal.pone.0169024. PMC5234772 28085888

[27] Cho NJ Park S Islam MI , et al. Long-term effect of medium cut-off dialyzer on middle uremic toxins and cell-free hemoglobin. PLoS ONE. 2019;14:e0220448. doi:10.1371/journal.pone.0220448. PMC6660073 31348802

[28] Yeter HH Korucu B Akcay OF Derici K Derici U Arinsoy T. Effects of medium cut-off dialysis membranes on inflammation and oxidative stress in patients on maintenance hemodialysis. Int Urol Nephrol. 2020;52(9):1779-1789. doi:10.1007/s11255-020-02562-3. 32661626

[29] Albrizio P Costa S Foschi A , et al. New medium cut-off membrane vs online hemodiafiltration in clearance of middle molecules. preliminary results from our centre. Nephrol Dial Transplant. 2018;33(suppl 1):i193. doi:10.1093/ndt/gfy104.FP466.

[30] Baharani J Barrios B Hopkins D , et al. UK clinical experiences of a new expanded haemodialysis therapy with a novel medium cut-off dialyser. J Amer Soc Nephrol. 2017;28:875.

[31] Bolton S Gair R Nilsson L-G , et al. Clinical assessment of dialysis recovery time and symptom burden: impact of switching hemodialysis therapy mode. Patient Related Outcome Measures. 2021;12:315-321. doi:10.2147/prom.s325016. 34764715 PMC8575372

[32] D’Achiardi R Zuñiga E Molano A , et al. P1063 Performance of medium cut-off dialyzers in expanded hemodialysis patients in Colombia. Nephrology Dialysis Transplantation. 2020;35. doi:10.1093/ndt/gfaa142.P1063.

[33] García-Prieto A Vega A Linares T , et al. Evaluation of the efficacy of a medium cut-off dialyser and comparison with other high-flux dialysers in conventional haemodialysis and online haemodiafiltration. Clin Kidney J. 2018;11(5):742-746. doi:10.1093/ckj/sfy004. 30288272 PMC6165747

[34] Gernone G Partipilo F Detomaso F , et al. P1084 Long term evaluation of the expanded hemodialysis (hdx) on dialysis adequacy, anemia and quality of life. Nephrology Dialysis Transplantation. 2020;35. doi:10.1093/ndt/gfaa142.P1084.

[35] Kim TH Kim SH Kim TY , et al. Removal of large middle molecules via haemodialysis with medium cut-off membranes at lower blood flow rates: an observational prospective study. BMC Nephrol. 2019;21:2. doi:10.1186/s12882-019-1669-3. 31892319 PMC6937993

[36] Krishnasamy R Hawley CM Jardine MJ , et al. A trial evaluating mid cut-off value membrane clearance of albumin and light chains in hemodialysis patients: a safety device study. Blood Purif. 2020;49(4):468-478. doi:10.1159/000505567. 31968346

[37] Penny J Salerno FR Hur L McIntyre C. P1062 Expanded dialysis (HDX): is there an impact on patient reported symptom? Nephrol Dial Transplant. 2020;35. doi:10.1093/ndt/gfaa142.P1062.

[38] Cozzolino M Magagnoli L Ciceri P , et al. Medium cut-off (Theranova) dialyzer reduces the number of infections in hemodialysis patients: a prospective, cross-over study. Nephrol Dial Transplant. 2019;34 (suppl 1):a515. doi:10.1093/ndt/gfz103.SP464.

[39] Lindsay RM Heidenheim PA Nesrallah G Garg AX Suri R , Daily Hemodialysis Study Group London Health Sciences Centre. Minutes to recovery after a hemodialysis session: a simple health-related quality of life question that is reliable, valid, and sensitive to change. Clin J Am Soc Nephrol. 2006;1(5):952-959. doi:10.2215/CJN.00040106. 17699312

[40] Allen RP Picchietti D Hening WA , et al. Restless legs syndrome: diagnostic criteria, special considerations, and epidemiology. A report from the restless legs syndrome diagnosis and epidemiology workshop at the National Institutes of Health. Sleep Med. 2003;4(2):101-119. doi:10.1016/s1389-9457(03)00010-8. 14592341

[41] Kandi M Brignardello-Petersen R Couban R , et al. Effects of medium cut-off versus high-flux hemodialysis membranes on biomarkers: a systematic review and meta-mnalysis. Canadian Journal of Kidney Health and Disease. 2021;8. 10.1177/20543581211067090 PMC8777328 35070336

[42] Group FHNT Chertow GM Levin NW , et al. In-center hemodialysis six times per week versus three times per week. N Engl J Med. 2010;363:2287-2300. doi:10.1056/NEJMoa1001593. 21091062 PMC3042140

[43] National Institutes of Health NIoDaDaKD. United States Renal Data System. 2019 USRDS Annual Data Report: Epidemiology of kidney disease in the United States. Bethesda, MD: National Institute of Diabetes and Digestive and Kidney Diseases, 2019.

[44] Smyth B Haber A Trongtrakul K , et al. Representativeness of randomized clinical trial cohorts in end-stage kidney disease. JAMA Intern Med. 2019;179:1316-1324. doi:10.1001/jamainternmed.2019.1501. 31282924 PMC6618769

[45] Bunch-Barrera A Tamer-David LM Ardila-Celis F , et al. Impacto de un modelo de gestión de enfermedad en una población con tratamiento de diálisis en Colombia. Revista de la Facultad de Medicina. 2016;64:695. doi:10.15446/revfacmed.v64n4.54556.

[46] Yan G Shen JI Harford R , et al. Racial and ethnic variations in mortality rates for patients undergoing maintenance dialysis treated in us territories compared with the US 50 states. Clinical Journal of the American Society of Nephrology. 2020;15:101-108. doi:10.2215/cjn.03920319. 31857376 PMC6946070

[47] den Hoedt CH Grooteman MP Bots ML , et al. The effect of online hemodiafiltration on infections: results from the CONvective TRAnsport STudy. PLoS ONE. 2015;10(8):e0135908. doi:10.1371/journal.pone.0135908. PMC4546111 26288091

[48] Corporation BH . Study in End Stage Renal Disease (ESRD) Patients on Hemodialysis Comparing the Theranova Dialyzer to Hemodiafiltration. https://ClinicalTrials.gov/show/NCT03547336, 2018. Accessed December 7, 2021.

[49] Hospital SNU Center SM Hospital SSMs , et al. Cardiovascular Risk Comparison Between Expanded Hemodialysis Using Theranova and On-line Hemodiafiltration. https://ClinicalTrials.gov/show/NCT03448887. Published 2018. Accessed December 7, 2021.

[50] Ishiwatari A Yamamoto S Fukuma S Hasegawa T Wakai S Nangaku M. Changes in quality of life in older hemodialysis patients: a cohort study on dialysis outcomes and practice patterns. Am J Nephrol. 2020;51(8):650-658. doi:10.1159/000509309. 32739911 PMC7592938

[51] Ricardo AC Hacker E Lora CM , et al. Validation of the Kidney Disease Quality of Life Short Form 36 (KDQOL-36) US Spanish and English versions in a cohort of Hispanics with chronic kidney disease. Ethn Dis. 2013;23(2):202-209. 23530302 PMC3651651

[52] Cohen DE Lee A Sibbel S , et al. Use of the KDQOL-36™ for assessment of health-related quality of life among dialysis patients in the United States. BMC Nephrol. 2019;20. doi:10.1186/s12882-019-1295-0. PMC6444438 30935377

[53] Samsa G Edelman D Rothman ML Williams GR Lipscomb J Matchar D. Determining clinically important differences in health status measures. Pharmacoeconomics. 1999;15(2):141-155. doi:10.2165/00019053-199915020-00003. 10351188

[54] Suri RS Garg AX Chertow GM , et al. Frequent Hemodialysis Network (FHN) randomized trials: study design. Kidney Int. 2007;71(4):349-359. doi:10.1038/sj.ki.5002032. 17164834

[55] Mapes DL Lopes AA Satayathum S , et al. Health-related quality of life as a predictor of mortality and hospitalization: the Dialysis Outcomes and Practice Patterns Study (DOPPS). Kidney Int. 2003;64(1):339-349. doi:10.1046/j.1523-1755.2003.00072.x. 12787427

[56] Rayner HC Zepel L Fuller DS , et al. Recovery time, quality of life, and mortality in hemodialysis patients: the Dialysis Outcomes and Practice Patterns Study (DOPPS). Am J Kidney Dis. 2014;64(1):86-94. doi:10.1053/j.ajkd.2014.01.014. 24529994 PMC4069238

[57] Garg AX Suri RS Eggers P , et al. Patients receiving frequent hemodialysis have better health-related quality of life compared to patients receiving conventional hemodialysis. Kidney Int. 2017;91(3):746-754. doi:10.1016/j.kint.2016.10.033. 28094031 PMC5333984

[58] Awuah KT Afolalu BA Hussein UT Raducu RR Bekui AM Finkelstein FO. Time to recovery after a hemodialysis session: impact of selected variables. Clin Kidney J. 2013;6(6):595-598. doi:10.1093/ckj/sft120. 26069828 PMC4438368

[59] Ramey G Deschemin JC Durel B Canonne-Hergaux F Nicolas G Vaulont S. Hepcidin targets ferroportin for degradation in hepatocytes. Haematologica. 2010;95(3):501-504. doi:10.3324/haematol.2009.014399. 19773263 PMC2833082

[60] Panichi V Scatena A Rosati A , et al. High-volume online haemodiafiltration improves erythropoiesis-stimulating agent (ESA) resistance in comparison with low-flux bicarbonate dialysis: results of the REDERT study. Nephrol Dial Transplant. 2015;30(4):682-689. doi:10.1093/ndt/gfu345. 25385719

[61] Mouillet G Efficace F Thiery-Vuillemin A , et al. Investigating the impact of open label design on patient-reported outcome results in prostate cancer randomized controlled trials. Cancer Med. 2020;9(20):7363-7374. doi:10.1002/cam4.3335. 32846465 PMC7571808

