# Supplementary material for: Clinical Outcomes With Medium Cut-Off Versus High-Flux Hemodialysis Membranes: A Systematic Review and Meta-Analysis
Source: Can J Kidney Health Dis. 2022 Jan 21;9:20543581211067087. doi: 10.1177/20543581211067087 (PMC8785433; doi:10.1177/20543581211067087)
Supplement: sj-docx-2-cjk-10.1177_20543581211067087 – Supplemental material for Clinical Outcomes With Medium Cut-Off Versus High-Flux Hemodialysis Membranes: A Systematic Review and Meta-Analysis [file sj-docx-2-cjk-10.1177_20543581211067087.docx]

# Appendix B – Detailed Methods

Protocol and registration

We registered our protocol (Appendix A) with the Prospero Register of Systematic Reviews (registration number CRD42020204636). We prepared this manuscript in accordance with the PRISMA guideline.^1^ We present abbreviated methods in the main manuscript and provide further details in this Appendix.

Eligibility criteria

Types of studies

We sought to include randomized studies (parallel arm and crossover), nonrandomized studies of interventions including cohort studies and before-after designs, as well as systematic reviews. We placed no restrictions on language. We limited the search timeframe to begin in 2015 to coincide with the first-ever published reports describing the intervention.

Types of participants

We included studies that enrolled adult patients (>18 years) with end-stage renal disease (ESRD) receiving maintenance hemodialysis. We excluded patients with acute kidney injury and those admitted to hospital.

Types of interventions

The only eligible intervention was a novel medium cutoff polyarylethersulfone /polyvinylpyrrolidone membrane with narrow pore size distribution (Theranova 400/500, Baxter Healthcare, Deerfield, IL, USA) and related prototypes previously owned by Gambro (Lund, Sweden). We excluded studies of high cutoff and “super high-flux” membranes. Eligible comparators were limited to high-flux membranes used in hemodialysis applications; we excluded studies in which the only comparators were hemofiltration/hemodiafiltration.

Types of outcomes

Primary efficacy outcomes included mortality, hospitalization, quality of life, symptoms, and other patient-reported outcome measures. Primary safety outcomes focused on hypothetical deficiency states resulting from nonselective protein and other large molecule removal potentially leading to impaired immunity or hypercoagulability. These included extracorporeal circuit and access thrombosis, infection, and albumin depletion.

Secondary/physiological outcomes included laboratory-based measures of β-2-microglobulin, myoglobin, λ- and κ-free light chains, IL-6, TNF-α, C-reactive protein, and protein-bound solutes. Where available, we included predialysis levels (reflecting the post-rebound steady state), reduction ratios, and clearance, or removal as measured through direct dialysate quantitation and/or blood-side clearance.

Information sources

We ran our primary search strategy in five databases (MEDLINE, EMBASE, CINAHL, Cochrane Library, and Web of Science), without restricting by language or study design, limiting by date to capture results since 2015. Grey literature sources included conference abstracts from pre-specified meetings (listed in protocol, Appendix A) to 2017. We also obtained a database of relevant articles compiled by the manufacturer, which included a comprehensive listing of conference abstracts, as well as full-text manuscripts reviewed and accepted for publication in peer-reviewed journals. We cross-referenced our primary search against this database for added sensitivity.

Search

Search concepts used by our information specialist (RC) were hemodialysis and medium cut-off membranes. Synonyms for each concept were combined using the OR operator, then the concepts were combined using the AND operator. The search strategy is in Appendix C.

Study selection

We imported citations into EndNote 9.3 for de-duplication then uploaded them to the DistillerSR online systematic review software platform for screening. For primary search results, we used pilot-tested title and abstract and full-text screening forms and screened all reports in duplicate resolving conflicts through discussion. Grey literature sources were screened by one reviewer. Where we identified multiple reports of the same study population, we use the most recent, comprehensive, or peer-reviewed report for data extraction.

Data collection process

Each reviewer extracted data independently into standard forms with independent verification by second reviewer.

Data items

We extracted key variables across the following categories: methods (design, setting), participant characteristics (demographics, eligibility criteria), characteristics of interventions, risk of bias evaluation criteria, patient disposition, and outcomes (including counts, rates, measures of central tendency and dispersion, and statistical significance).

When studies reported measures at multiple time points, we used the last available value (representing the longest possible follow-up) for meta-analysis. We extracted counts of patients with one or more hospitalization events rather than total number of hospitalizations per group to avoid double counting. For studies reporting death or hospitalization, we only considered those with a minimum of 10 weeks of follow-up for meta-analysis, excluding those with shorter follow-up from pooled estimates for these outcomes.

Studies that reported the reduction ratio for any solute with concentration C used the following formula:

The final post-dialysis solute concentration (C*_post_*) was corrected for the degree of hemoconcentration and the volume of distribution (approximate extracellular volume) according to Bergström and Wehle:^2^

To give C*_post-corr_*, where BW represents body weight pre- and post-dialysis.

Risk of bias in individual studies

Two reviewers independently used the Cochrane Risk of Bias tools version 2 for randomized studies^3^, crossover trials^4^, and the ROBINS-I tool for nonrandomized studies^5^. ROBINS-I includes 7 domains that compare each nonrandomized study to an ‘ideal’ pragmatic trial, enabling direct comparisons of the certainty of evidence arising from randomized and nonrandomized studies for a given outcome.

We anticipated significant potential carryover effects such as a sustained reduction in large middle-molecule concentrations after treatment with Theranova. However, since this effect would have biased effect estimates towards the null, we did not rate down for risk of bias based on the duration of washout periods in crossover trials.

Summary measures

For continuous variables, we extracted change scores and their corresponding standard errors (SEs) or *P* values, where available, and used *P* values to impute the SE for change where required, using the following formula:

$$SE=\sqrt{\frac{\left( N_{1}-1 \right)S_{1}^{2}+(N_{2}-1)S_{2}^{2}}{N_{1}+N_{2}-2})(\frac{1}{N_{1}}+\frac{1}{N_{2}})}$$

Where this was infeasible, we collected final values and their corresponding measures of dispersion and planned to meta-analyze change scores and final values as subgroups and pool these estimates if appropriate.

We planned to use the patient as the unit of analysis. For randomized crossover trials, we extracted treatment effect estimates reported as paired analyses where possible.

When studies did not report a standard deviation for given value, we imputed it using the mean coefficient of variation from the other studies reporting a mean and SD for that measure.

For outcomes that were reported using different units of measurement (*e.g.,* erythropoiesis resistance index and C-reactive protein), we calculated the standardized mean difference (SMD) and related SE estimate, using the following formulae ^6^:

$$SMD=\frac{X_{1}-X_{2}}{S_{pooled}}$$

$$S_{pooled}=\sqrt{\frac{\left( N_{1}-1 \right)S_{1}^{2}+(N_{2}-1)S_{2}^{2}}{N_{1}+N_{2}-2})}$$

$$\mathrm{SE}_{SMD}=\sqrt{\frac{N_{1}+N_{2}}{N_{1}N_{2}}+\frac{{SMD}^{2}}{N_{1}+N_{2}-2}}$$

For all equations:

$X_{1}$: Mean in high-flux HD group

$X_{2}$: Mean in MCO group

$N_{1}$: sample size in high-flux HD group

$N_{2}$: sample size in MCO group

$S_{1}^{2}$: variance in high-flux HD group

$S_{2}^{2}$: variance in MCO group

Synthesis of results

We used the generic inverse variance method to pool continuous data using mean differences or SMDs and corresponding SE estimates. For count data including mortality, hospitalization, and infection, we calculated total follow-up in each study arm (in patient-days) and calculated risk ratios and corresponding standard error estimates. We calculated odds ratios and corresponding SEs for dichotomous outcomes. In all instances, we planned to use random-effect models, reverting to fixed-effects models where only 2 studies were available for pooling and where a random-effects model resulted in inappropriately large weighting of a small study. We pooled randomized trials and observational studies separately. Where the quality of evidence/certainty was identical for randomized trials and observational studies for a given outcome, and where heterogeneity was undetected or low, we pooled effect estimates from both bodies of evidence. In most instances, this was done to improve precision where it would have otherwise been low with each estimate reported separately.

While extracting data, we realized that many patient-reported outcome measures were available for meta-analysis. In order to avoid any potential bias in grouping these outcomes (given that all members of the review team had seen some or all of the extracted data), we used the approach proposed by Johnston et al. and provided a list of available measures (instruments and subscales) to an independent collaborator to generate appropriate groupings of measures in a blinded manner ^7^.

We planned to use the *I*^2^ statistic to explore heterogeneity through subgroup analysis with prespecified subgroups defined by study duration (short versus long), publication type (peer-reviewed versus conference abstracts), funding source, and based on other study design characteristics, such as availability of change scores versus final values and presence versus absence of blinding of participants providing patient-reported outcome measures.

When studies met eligibility criteria for population, intervention, and comparator but did not report any prespecified outcomes, or were not amenable to meta-analysis, we extracted and summarized their key findings using standard narrative synthesis techniques.

Risk of bias across studies

Where possible, we used funnel plots to assess for publication bias.

Certainty Assessment

We assessed the certainty of evidence separately for each outcome using GRADE and summarized these assessments in a Summary of Findings Table using GRADEpro: <https://gdt.gradepro.org/app/#projects>^8^. Certainty was rated as very low, low, moderate, or high. Effect estimates for randomized and nonrandomized studies started with high certainty and were downgraded 1 or 2 levels for risk of bias^9, 10^, inconsistency^11^, indirectness^12^, imprecision^13^, or publication bias^14^. We appraised certainty on an outcome-by-outcome basis, considering the specific studies contributing to each effect estimate. In doing so, we considered the relative contribution (weight) of each study when rating the risk of bias across studies. We rated up for large effects, dose-response, and opposing residual confounding bias in nonrandomized studies^15^. We assessed imprecision for dichotomous outcomes using nomograms for optimal information size. For continuous outcomes, we estimated optimal information size using sample size calculators for paired and unpaired comparisons as appropriate for the observed effect size, using β=0.8 and α=0.05^13^. We calculated absolute treatment effects based on control event rates in studies included for each outcome^16^. We used validated algorithms to produce informative qualitative statements describing review findings and used these phrases throughout this report (Table 2, column labelled “What Happens”)^17^.

## REFERENCES

1. Liberati A, Altman DG, Tetzlaff J, et al. The PRISMA Statement for Reporting Systematic Reviews and Meta-Analyses of Studies That Evaluate Health Care Interventions: Explanation and Elaboration. *PLoS Medicine* 2009; 6: e1000100. DOI: 10.1371/journal.pmed.1000100.

2. Bergström J and Wehle B. NO CHANGE IN CORRECTED β2-MICROGLOBULIN CONCENTRATION AFTER CUPROPHANE HAEMODIALYSIS. 1987; 329: 628-629. DOI: 10.1016/s0140-6736(87)90266-2.

3. Sterne JAC, Savovic J, Page MJ, et al. RoB 2: a revised tool for assessing risk of bias in randomised trials. *BMJ* 2019; 366: l4898. 2019/08/30. DOI: 10.1136/bmj.l4898.

4. JPT H, Tianjing L and Sterne J. Revised Cochrane risk of bias tool for randomized trials (RoB 2) Additional considerations for crossover trials, <https://www.riskofbias.info/welcome/rob-2-0-tool/rob-2-for-crossover-trials> (2021, accessed April 1, 2021).

5. Sterne JA, Hernán MA, Reeves BC, et al. ROBINS-I: a tool for assessing risk of bias in non-randomised studies of interventions. *BMJ* 2016: i4919. DOI: 10.1136/bmj.i4919.

6. Higgins JPT, Thomas J, Chandler J, et al. *Cochrane Handbook for Systematic Reviews of Interventions version 6.0 (updated July 2019)*. Cochrane, 2019.

7. Johnston BC, Patrick DL, Busse JW, et al. Patient-reported outcomes in meta-analyses--Part 1: assessing risk of bias and combining outcomes. *Health Qual Life Outcomes* 2013; 11: 109. 2013/07/03. DOI: 10.1186/1477-7525-11-109.

8. Guyatt GH, Oxman AD, Santesso N, et al. GRADE guidelines: 12. Preparing summary of findings tables-binary outcomes. *J Clin Epidemiol* 2013; 66: 158-172. 2012/05/23. DOI: 10.1016/j.jclinepi.2012.01.012.

9. Guyatt GH, Oxman AD, Vist G, et al. GRADE guidelines: 4. Rating the quality of evidence--study limitations (risk of bias). *J Clin Epidemiol* 2011; 64: 407-415. 2011/01/21. DOI: 10.1016/j.jclinepi.2010.07.017.

10. Schunemann HJ, Cuello C, Akl EA, et al. GRADE guidelines: 18. How ROBINS-I and other tools to assess risk of bias in nonrandomized studies should be used to rate the certainty of a body of evidence. *J Clin Epidemiol* 2019; 111: 105-114. 2018/02/13. DOI: 10.1016/j.jclinepi.2018.01.012.

11. Guyatt GH, Oxman AD, Kunz R, et al. GRADE guidelines: 7. Rating the quality of evidence--inconsistency. *J Clin Epidemiol* 2011; 64: 1294-1302. 2011/08/02. DOI: 10.1016/j.jclinepi.2011.03.017.

12. Guyatt GH, Oxman AD, Kunz R, et al. GRADE guidelines: 8. Rating the quality of evidence--indirectness. *J Clin Epidemiol* 2011; 64: 1303-1310. 2011/08/02. DOI: 10.1016/j.jclinepi.2011.04.014.

13. Guyatt GH, Oxman AD, Kunz R, et al. GRADE guidelines 6. Rating the quality of evidence--imprecision. *J Clin Epidemiol* 2011; 64: 1283-1293. 2011/08/16. DOI: 10.1016/j.jclinepi.2011.01.012.

14. Guyatt GH, Oxman AD, Montori V, et al. GRADE guidelines: 5. Rating the quality of evidence--publication bias. *J Clin Epidemiol* 2011; 64: 1277-1282. 2011/08/02. DOI: 10.1016/j.jclinepi.2011.01.011.

15. Guyatt GH, Oxman AD, Sultan S, et al. GRADE guidelines: 9. Rating up the quality of evidence. *J Clin Epidemiol* 2011; 64: 1311-1316. 2011/08/02. DOI: 10.1016/j.jclinepi.2011.06.004.

16. Newcombe RG and Bender R. Implementing GRADE: calculating the risk difference from the baseline risk and the relative risk. *Evid Based Med* 2014; 19: 6-8. 2013/08/24. DOI: 10.1136/eb-2013-101340.

17. Santesso N, Glenton C, Dahm P, et al. GRADE guidelines 26: informative statements to communicate the findings of systematic reviews of interventions. *J Clin Epidemiol* 2019 2019/11/13. DOI: 10.1016/j.jclinepi.2019.10.014.
