# Supplementary material for: Clinical Outcomes With Medium Cut-Off Versus High-Flux Hemodialysis Membranes: A Systematic Review and Meta-Analysis
Source: Can J Kidney Health Dis. 2022 Jan 21;9:20543581211067087. doi: 10.1177/20543581211067087 (PMC8785433; doi:10.1177/20543581211067087)
Supplement: sj-docx-3-cjk-10.1177_20543581211067087 – Supplemental material for Clinical Outcomes With Medium Cut-Off Versus High-Flux Hemodialysis Membranes: A Systematic Review and Meta-Analysis [file sj-docx-3-cjk-10.1177_20543581211067087.docx]

# Appendix C - Summary of search results and strategy

We will search EMBASE, MEDLINE, CINAHL, CDSR, and ISI Web of Science from 2015 to present

July 16, 2020

| MEDLINE | 320 |
| --- | --- |
| EMBASE | 738 |
| CINAHL | 69 |
| CDSR | 171 |
| WoS | 500 |
| Subtotal | 1798 |
| -dupes | -595 |
| Total | 1203 |

MEDLINE

Database: OVID Medline Epub Ahead of Print, In-Process & Other Non-Indexed Citations, Ovid MEDLINE(R) Daily and Ovid MEDLINE(R) 1946 to Present

Search Strategy:

--------------------------------------------------------------------------------

1 renal dialysis/ or hemodiafiltration/ (92729)

2 Kidney Failure, Chronic/ (93331)

3 (dialysis or h?emodialysis or diafiltration or h?emodiafiltration).mp. [mp=title, abstract, original title, name of substance word, subject heading word, floating sub-heading word, keyword heading word, organism supplementary concept word, protocol supplementary concept word, rare disease supplementary concept word, unique identifier, synonyms] (185491)

4 or/1-3 (225923)

5 Theranova.mp. (11)

6 MCO.mp. [mp=title, abstract, original title, name of substance word, subject heading word, floating sub-heading word, keyword heading word, organism supplementary concept word, protocol supplementary concept word, rare disease supplementary concept word, unique identifier, synonyms] (1114)

7 expanded hemodialysis.mp. (17)

8 HDx.mp. (830)

9 High-Retention-Onset Membranes.mp. (4)

10 ((medium or mid or middle) and (cut-off or cut off)).mp. (24612)

11 HRO.mp. [mp=title, abstract, original title, name of substance word, subject heading word, floating sub-heading word, keyword heading word, organism supplementary concept word, protocol supplementary concept word, rare disease supplementary concept word, unique identifier, synonyms] (123)

12 (((medium or middle*) adj3 molecul*) and membrane*).mp. [mp=title, abstract, original title, name of substance word, subject heading word, floating sub-heading word, keyword heading word, organism supplementary concept word, protocol supplementary concept word, rare disease supplementary concept word, unique identifier, synonyms] (641)

13 ((albumin or microglobulin or myoglobulin) and (dialysis membrane* or dialyzer or dialyzate)).mp. [mp=title, abstract, original title, name of substance word, subject heading word, floating sub-heading word, keyword heading word, organism supplementary concept word, protocol supplementary concept word, rare disease supplementary concept word, unique identifier, synonyms] (627)

14 or/5-13 (27777)

15 4 and 14 (1423)

16 limit 15 to yr="2015 -Current" (320)

EMBASE (Ovid)

Database: Embase <1974 to 2020 July 15>

Search Strategy:

--------------------------------------------------------------------------------

1 hemodialysis/ (107190)

2 hemodiafiltration/ (3011)

3 chronic kidney failure/ (92123)

4 end stage renal disease/ (31501)

5 (dialysis or h?emodialysis or diafiltration or h?emodiafiltration).mp. [mp=title, abstract, heading word, drug trade name, original title, device manufacturer, drug manufacturer, device trade name, keyword, floating subheading word, candidate term word] (257023)

6 or/1-5 (329356)

7 Theranova.mp. (43)

8 MCO.mp. [mp=title, abstract, heading word, drug trade name, original title, device manufacturer, drug manufacturer, device trade name, keyword, floating subheading word, candidate term word] (1437)

9 expanded hemodialysis.mp. (27)

10 HDx.mp. (2389)

11 High-Retention-Onset Membranes.mp. (4)

12 ((medium or mid or middle) and (cut-off or cut off)).mp. (14051)

13 HRO.mp. [mp=title, abstract, heading word, drug trade name, original title, device manufacturer, drug manufacturer, device trade name, keyword, floating subheading word, candidate term word] (177)

14 (((medium or middle*) adj3 molecul*) and membrane*).mp. [mp=title, abstract, heading word, drug trade name, original title, device manufacturer, drug manufacturer, device trade name, keyword, floating subheading word, candidate term word] (811)

15 ((albumin or microglobulin or myoglobulin) and (dialysis membrane* or dialyzer or dialyzate)).mp. [mp=title, abstract, heading word, drug trade name, original title, device manufacturer, drug manufacturer, device trade name, keyword, floating subheading word, candidate term word] (1479)

16 or/7-15 (19983)

17 6 and 16 (2192)

18 limit 17 to yr="2015 -Current" (738)

CINAHL (EBSCO)

| **#** | **Query** | **Results** |
| --- | --- | --- |
| S17 | S16 Limiters - Published Date: 20150101-20201231 Expanders | 69 |
| S16 | S5 AND S15 | 157 |
| S15 | S6 OR S7 OR S8 OR S9 OR S10 OR S11 OR S12 OR S13 OR S14 | 7299 |
| S14 | TX ((albumin or microglobulin or myoglobulin) and (dialysis membrane* or dialyzer or dialyzate)). | 46 |
| S13 | TX (((medium or middle*) N3 molecul*) and membrane*) | 37 |
| S12 | TX HRO | 79 |
| S11 | TX ((medium or mid or middle) and (cut-off or cut off)) | 6484 |
| S10 | TX High-Retention-Onset Membranes | 0 |
| S9 | TX HDx | 14 |
| S8 | TX expanded hemodialysis | 2 |
| S7 | TX MCO | 653 |
| S6 | TX theranova | 1 |
| S5 | S1 OR S2 OR S3 OR S4 | 50,232 |
| S4 | TX haemodialysis or haemodiafiltration | 23,112 |
| S3 | dialysis or hemodialysis or diafiltration or hemodiafiltration | 38,140 |
| S2 | (MH "Kidney Failure, Chronic") | 23,162 |
| S1 | (MH "Hemodialysis") OR (MH "Hemodiafiltration") | 16,849 |

Cochrane Library (Wiley)

Search Name: MCO membrane

Date Run: 16/07/2020 21:33:10

Comment:

ID Search Hits

#1 MeSH descriptor: [Renal Dialysis] explode all trees 5059

#2 MeSH descriptor: [Hemodiafiltration] explode all trees 239

#3 MeSH descriptor: [Kidney Failure, Chronic] explode all trees 4633

#4 dialysis or h?emodialysis or diafiltration or h?emodiafiltration 19819

#5 #1 or #2 or #3 or #4 21162

#6 theranova 28

#7 MCO 157

#8 HDx 21

#9 High-Retention-Onset Membranes 1

#10 ((medium or mid or middle) and (cut-off or cut off)) 2144

#11 HRO 7

#12 (((medium or middle*) NEAR/3 molecul*) and membrane*) 104

#13 ((albumin or microglobulin or myoglobulin) and (dialysis membrane* or dialyzer or dialyzate)) 504

#14 #6 or #7 or #8 or #9 or #10 or #11 or #12 or #13 2790

#15 #5 and #14 with Publication Year from 2015 to 2020, in Trials 171

Web of Science (Clarivate)

|  |  |  |  |  |  |
| --- | --- | --- | --- | --- | --- |
| # 12 | [**500**](http://apps.webofknowledge.com/summary.do?product=WOS&doc=1&qid=24&SID=7FNbMZ6Y5aQyPOuaFPf&search_mode=AdvancedSearch&update_back2search_link_param=yes) | #11  *Indexes=SCI-EXPANDED, SSCI, A&HCI, CPCI-S, CPCI-SSH, BKCI-S, BKCI-SSH, ESCI, CCR-EXPANDED, IC Timespan=2015-2020* | [Edit](http://apps.webofknowledge.com/WOS_AdvancedSearch_input.do?product=WOS&SID=7FNbMZ6Y5aQyPOuaFPf&search_mode=AdvancedSearch&replaceSetId=12&editState=init) | 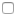 | 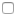 |
|  | | | | | |
| # 11 | [**2,436**](http://apps.webofknowledge.com/summary.do?product=WOS&doc=1&qid=22&SID=7FNbMZ6Y5aQyPOuaFPf&search_mode=CombineSearches&update_back2search_link_param=yes) | #10 AND #1  *Indexes=SCI-EXPANDED, SSCI, A&HCI, CPCI-S, CPCI-SSH, BKCI-S, BKCI-SSH, ESCI, CCR-EXPANDED, IC Timespan=All years* | [Edit](http://apps.webofknowledge.com/WOS_AdvancedSearch_input.do?product=WOS&SID=7FNbMZ6Y5aQyPOuaFPf&search_mode=AdvancedSearch&replaceSetId=11&editState=init) | 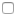 | 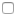 |
|  | | | | | |
| # 10 | [**10,136**](http://apps.webofknowledge.com/summary.do?product=WOS&doc=1&qid=21&SID=7FNbMZ6Y5aQyPOuaFPf&search_mode=CombineSearches&update_back2search_link_param=yes) | #9 OR #8 OR #7 OR #6 OR #5 OR #4 OR #3 OR #2  *Indexes=SCI-EXPANDED, SSCI, A&HCI, CPCI-S, CPCI-SSH, BKCI-S, BKCI-SSH, ESCI, CCR-EXPANDED, IC Timespan=All years* | [Edit](http://apps.webofknowledge.com/WOS_AdvancedSearch_input.do?product=WOS&SID=7FNbMZ6Y5aQyPOuaFPf&search_mode=AdvancedSearch&replaceSetId=10&editState=init) | 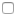 | 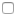 |
|  | | | | | |
| # 9 | [**2,233**](http://apps.webofknowledge.com/summary.do?product=WOS&doc=1&qid=18&SID=7FNbMZ6Y5aQyPOuaFPf&search_mode=GeneralSearch&update_back2search_link_param=yes) | **TOPIC:**  (((albumin or microglobulin or myoglobulin)  and  (dialysis membrane* or dialyzer or dialyzate) ))  *Indexes=SCI-EXPANDED, SSCI, A&HCI, CPCI-S, CPCI-SSH, BKCI-S, BKCI-SSH, ESCI, CCR-EXPANDED, IC Timespan=All years* | [Edit](http://apps.webofknowledge.com/WOS_AdvancedSearch_input.do?product=WOS&SID=7FNbMZ6Y5aQyPOuaFPf&search_mode=AdvancedSearch&replaceSetId=9&editState=init) | 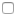 | 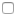 |
|  | | | | | |
| # 8 | [**845**](http://apps.webofknowledge.com/summary.do?product=WOS&doc=1&qid=14&SID=7FNbMZ6Y5aQyPOuaFPf&search_mode=GeneralSearch&update_back2search_link_param=yes) | **TOPIC:**  ((((medium or middle*)  NEAR/3  molecul*)  and  membrane*))  *Indexes=SCI-EXPANDED, SSCI, A&HCI, CPCI-S, CPCI-SSH, BKCI-S, BKCI-SSH, ESCI, CCR-EXPANDED, IC Timespan=All years* | [Edit](http://apps.webofknowledge.com/WOS_AdvancedSearch_input.do?product=WOS&SID=7FNbMZ6Y5aQyPOuaFPf&search_mode=AdvancedSearch&replaceSetId=8&editState=init) | 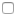 | 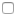 |
|  | | | | | |
| # 7 | [**293**](http://apps.webofknowledge.com/summary.do?product=WOS&doc=1&qid=13&SID=7FNbMZ6Y5aQyPOuaFPf&search_mode=GeneralSearch&update_back2search_link_param=yes) | **TOPIC:**  (HRO)  *Indexes=SCI-EXPANDED, SSCI, A&HCI, CPCI-S, CPCI-SSH, BKCI-S, BKCI-SSH, ESCI, CCR-EXPANDED, IC Timespan=All years* | [Edit](http://apps.webofknowledge.com/WOS_AdvancedSearch_input.do?product=WOS&SID=7FNbMZ6Y5aQyPOuaFPf&search_mode=AdvancedSearch&replaceSetId=7&editState=init) | 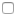 | 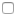 |
|  | | | | | |
| # 6 | [**4,753**](http://apps.webofknowledge.com/summary.do?product=WOS&doc=1&qid=12&SID=7FNbMZ6Y5aQyPOuaFPf&search_mode=GeneralSearch&update_back2search_link_param=yes) | **TOPIC:**  (((medium or mid or middle)  and  (cut-off or cut off) ))  *Indexes=SCI-EXPANDED, SSCI, A&HCI, CPCI-S, CPCI-SSH, BKCI-S, BKCI-SSH, ESCI, CCR-EXPANDED, IC Timespan=All years* | [Edit](http://apps.webofknowledge.com/WOS_AdvancedSearch_input.do?product=WOS&SID=7FNbMZ6Y5aQyPOuaFPf&search_mode=AdvancedSearch&replaceSetId=6&editState=init) | 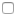 | 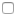 |
|  | | | | | |
| # 5 | [**13**](http://apps.webofknowledge.com/summary.do?product=WOS&doc=1&qid=11&SID=7FNbMZ6Y5aQyPOuaFPf&search_mode=GeneralSearch&update_back2search_link_param=yes) | **TOPIC:**  (High-Retention-Onset Membranes)  *Indexes=SCI-EXPANDED, SSCI, A&HCI, CPCI-S, CPCI-SSH, BKCI-S, BKCI-SSH, ESCI, CCR-EXPANDED, IC Timespan=All years* | [Edit](http://apps.webofknowledge.com/WOS_AdvancedSearch_input.do?product=WOS&SID=7FNbMZ6Y5aQyPOuaFPf&search_mode=AdvancedSearch&replaceSetId=5&editState=init) | 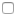 | 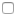 |
|  | | | | | |
| # 4 | [**885**](http://apps.webofknowledge.com/summary.do?product=WOS&doc=1&qid=10&SID=7FNbMZ6Y5aQyPOuaFPf&search_mode=GeneralSearch&update_back2search_link_param=yes) | **TOPIC:**  (HDx)  *Indexes=SCI-EXPANDED, SSCI, A&HCI, CPCI-S, CPCI-SSH, BKCI-S, BKCI-SSH, ESCI, CCR-EXPANDED, IC Timespan=All years* | [Edit](http://apps.webofknowledge.com/WOS_AdvancedSearch_input.do?product=WOS&SID=7FNbMZ6Y5aQyPOuaFPf&search_mode=AdvancedSearch&replaceSetId=4&editState=init) | 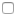 | 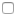 |
|  | | | | | |
| # 3 | [**1,384**](http://apps.webofknowledge.com/summary.do?product=WOS&doc=1&qid=9&SID=7FNbMZ6Y5aQyPOuaFPf&search_mode=GeneralSearch&update_back2search_link_param=yes) | **TOPIC:**  (MCO)  *Indexes=SCI-EXPANDED, SSCI, A&HCI, CPCI-S, CPCI-SSH, BKCI-S, BKCI-SSH, ESCI, CCR-EXPANDED, IC Timespan=All years* | [Edit](http://apps.webofknowledge.com/WOS_AdvancedSearch_input.do?product=WOS&SID=7FNbMZ6Y5aQyPOuaFPf&search_mode=AdvancedSearch&replaceSetId=3&editState=init) | 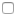 | 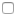 |
|  | | | | | |
| # 2 | [**13**](http://apps.webofknowledge.com/summary.do?product=WOS&doc=1&qid=6&SID=7FNbMZ6Y5aQyPOuaFPf&search_mode=GeneralSearch&update_back2search_link_param=yes) | **TOPIC:**  (theranova)  *Indexes=SCI-EXPANDED, SSCI, A&HCI, CPCI-S, CPCI-SSH, BKCI-S, BKCI-SSH, ESCI, CCR-EXPANDED, IC Timespan=All years* | [Edit](http://apps.webofknowledge.com/WOS_AdvancedSearch_input.do?product=WOS&SID=7FNbMZ6Y5aQyPOuaFPf&search_mode=AdvancedSearch&replaceSetId=2&editState=init) | 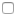 | 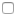 |
|  | | | | | |
| # 1 | [**172,918**](http://apps.webofknowledge.com/summary.do?product=WOS&doc=1&qid=5&SID=7FNbMZ6Y5aQyPOuaFPf&search_mode=GeneralSearch&update_back2search_link_param=yes) | **TOPIC:**  (dialysis or haemodialysis or hemodialysis or diafiltration or haemodiafiltration or hemodiafiltration)  *Indexes=SCI-EXPANDED, SSCI, A&HCI, CPCI-S, CPCI-SSH, BKCI-S, BKCI-SSH, ESCI, CCR-EXPANDED, IC Timespan=All years* | [Edit](http://apps.webofknowledge.com/WOS_AdvancedSearch_input.do?product=WOS&SID=7FNbMZ6Y5aQyPOuaFPf&search_mode=AdvancedSearch&replaceSetId=1&editState=init) |  |  |
