# Supplementary material for: Clinical Outcomes With Medium Cut-Off Versus High-Flux Hemodialysis Membranes: A Systematic Review and Meta-Analysis
Source: Can J Kidney Health Dis. 2022 Jan 21;9:20543581211067087. doi: 10.1177/20543581211067087 (PMC8785433; doi:10.1177/20543581211067087)
Supplement: sj-pptx-5-cjk-10.1177_20543581211067087 – Supplemental material for Clinical Outcomes With Medium Cut-Off Versus High-Flux Hemodialysis Membranes: A Systematic Review and Meta-Analysis [file sj-pptx-5-cjk-10.1177_20543581211067087.pptx]

## Slide 1
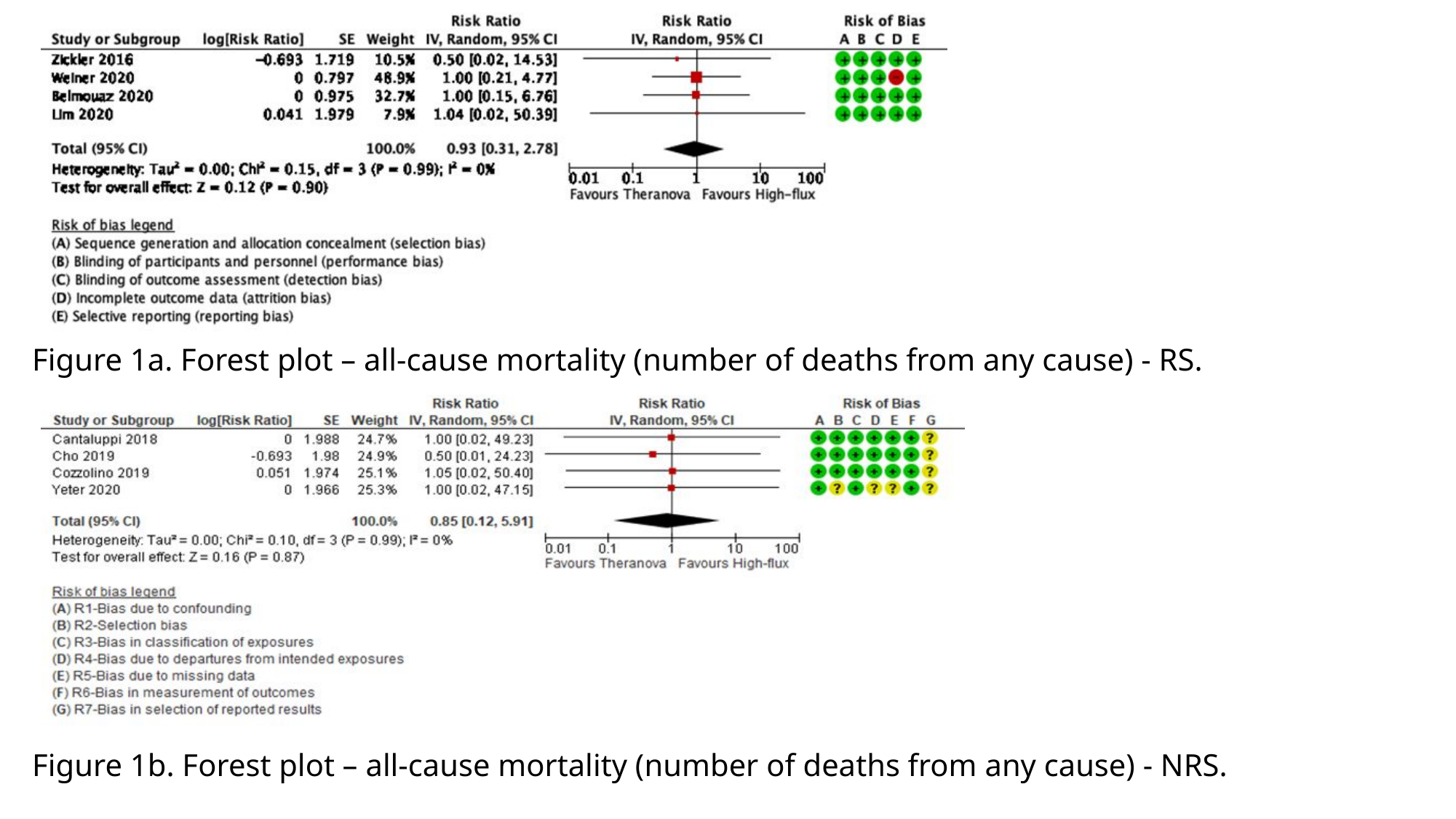

# Figure 1a. Forest plot – all-cause mortality (number of deaths from any cause) - RS.
Figure 1b. Forest plot – all-cause mortality (number of deaths from any cause) - NRS.

## Slide 2
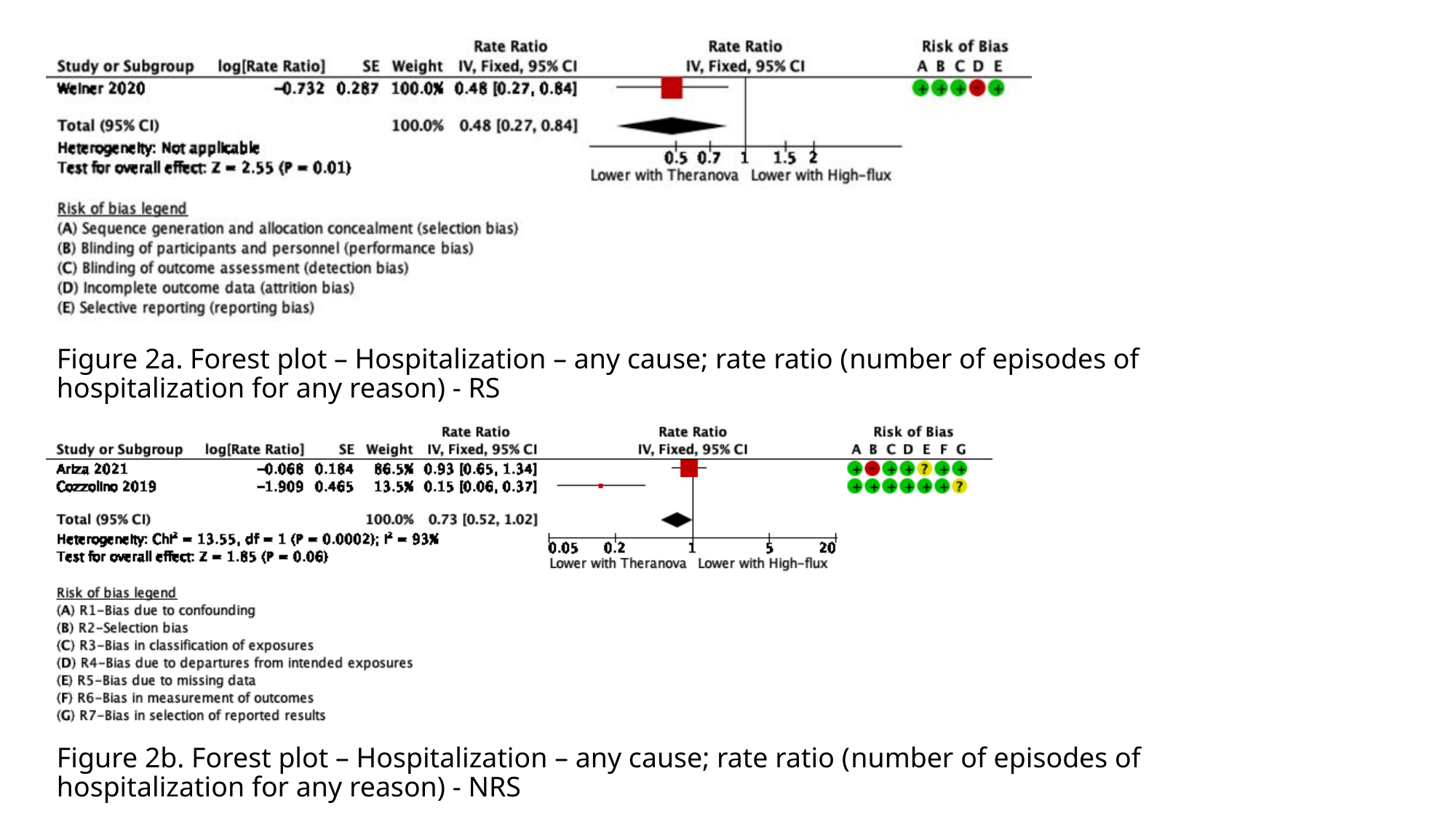

Figure 2a. Forest plot – Hospitalization – any cause; rate ratio (number of episodes of hospitalization for any reason) - RS
Figure 2b. Forest plot – Hospitalization – any cause; rate ratio (number of episodes of hospitalization for any reason) - NRS

## Slide 3
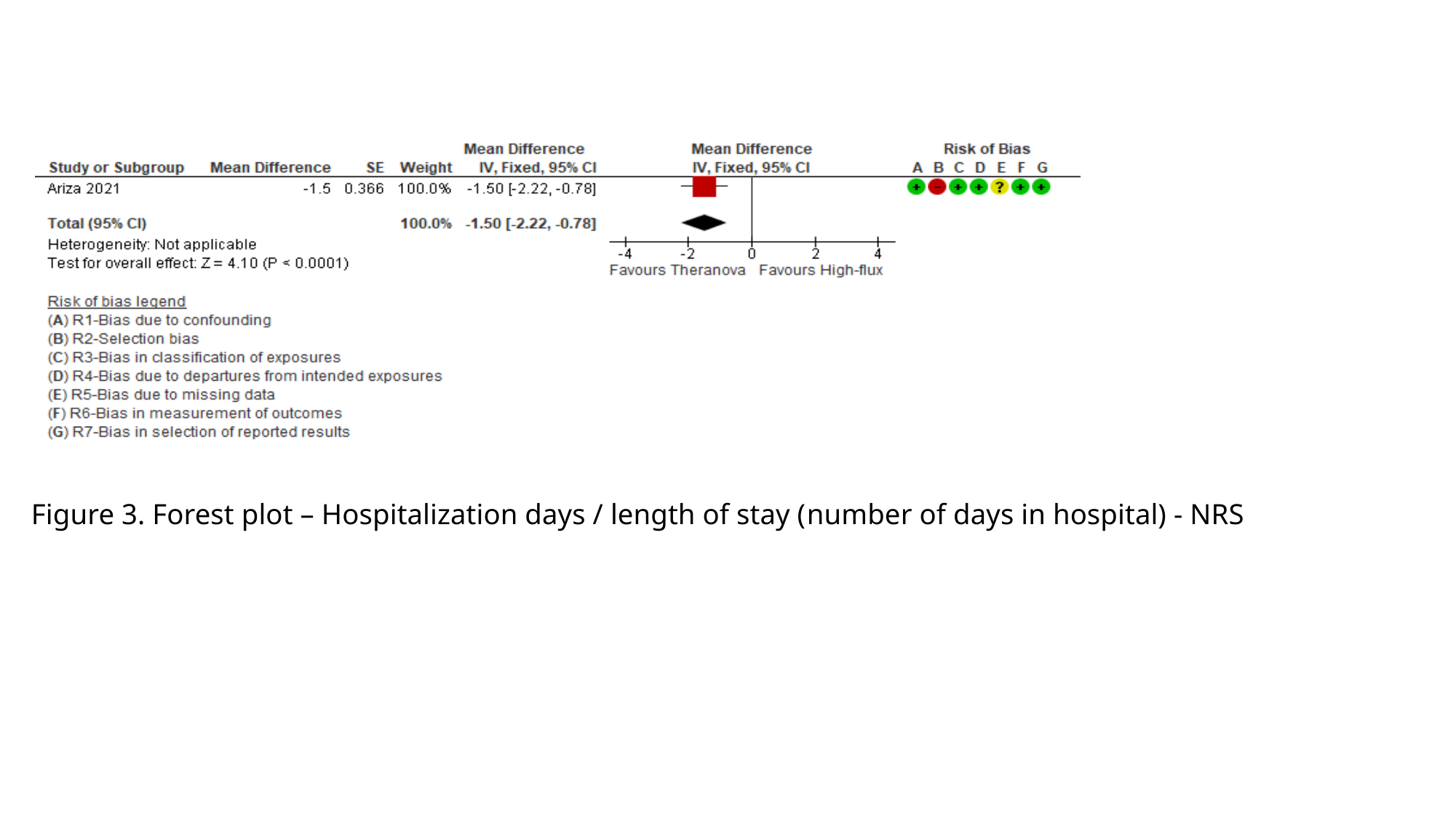

# Figure 3. Forest plot – Hospitalization days / length of stay (number of days in hospital) - NRS

## Slide 4
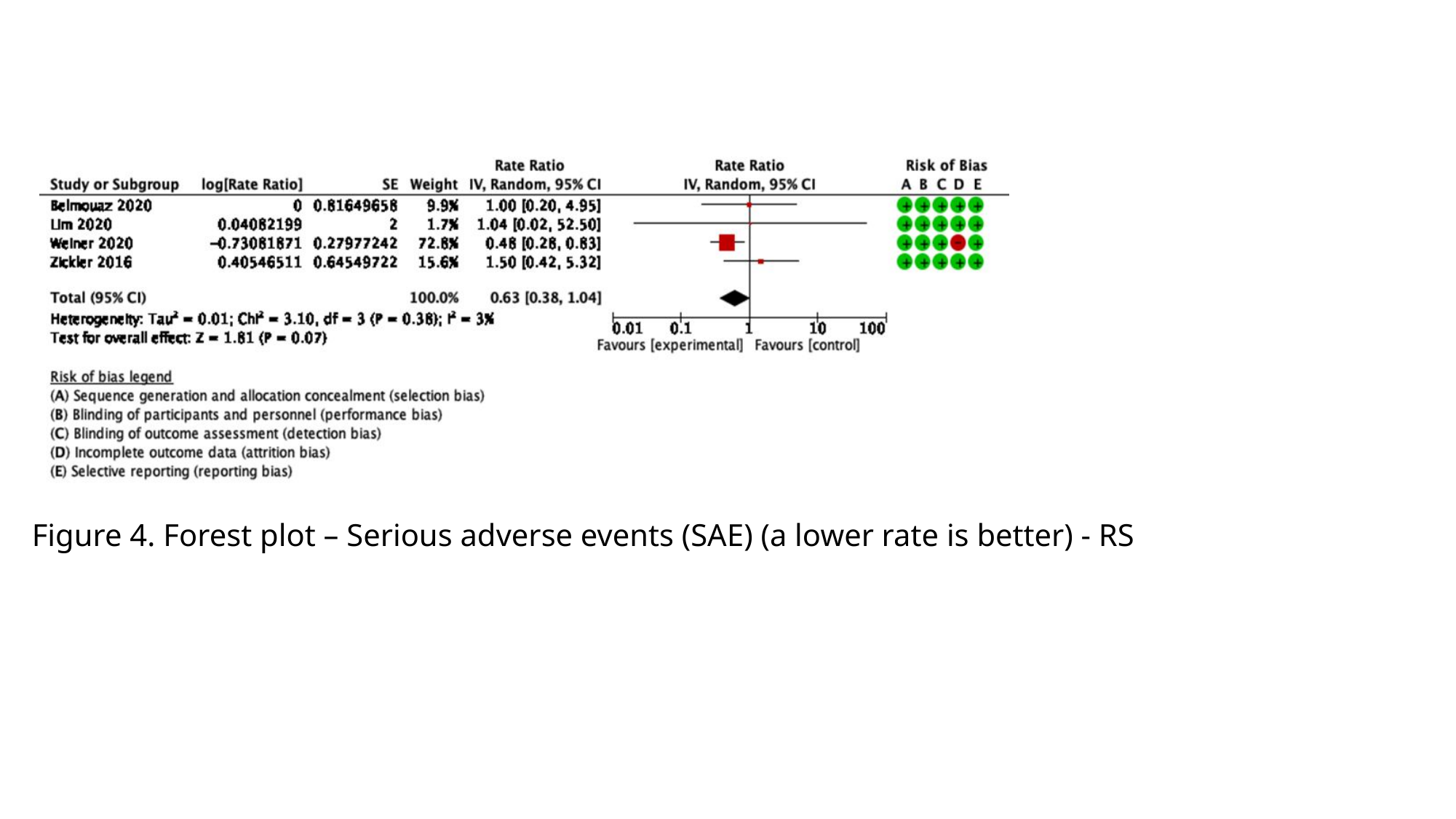

# Figure 4. Forest plot – Serious adverse events (SAE) (a lower rate is better) - RS

## Slide 5
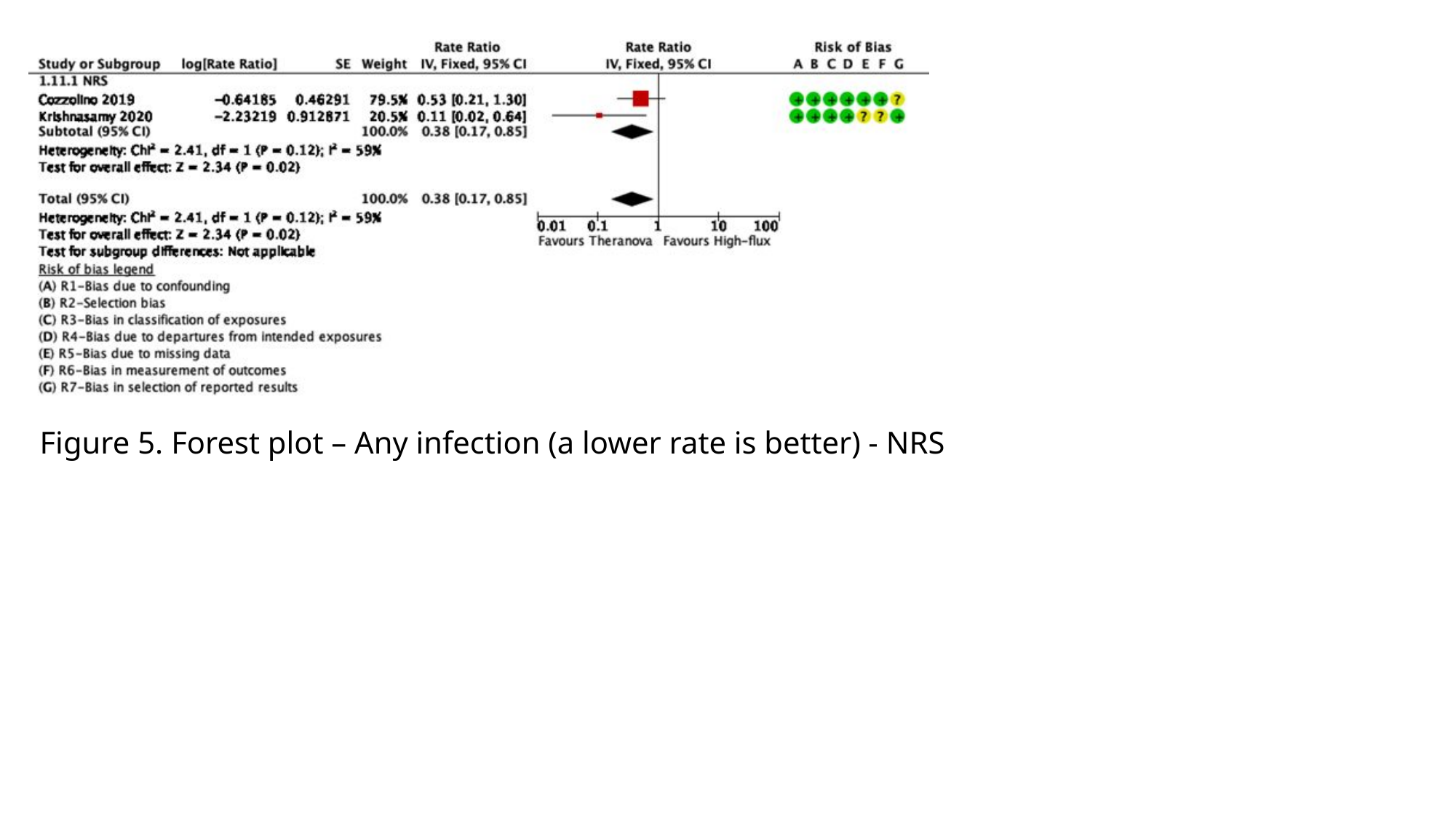

# Figure 5. Forest plot – Any infection (a lower rate is better) - NRS

## Slide 6
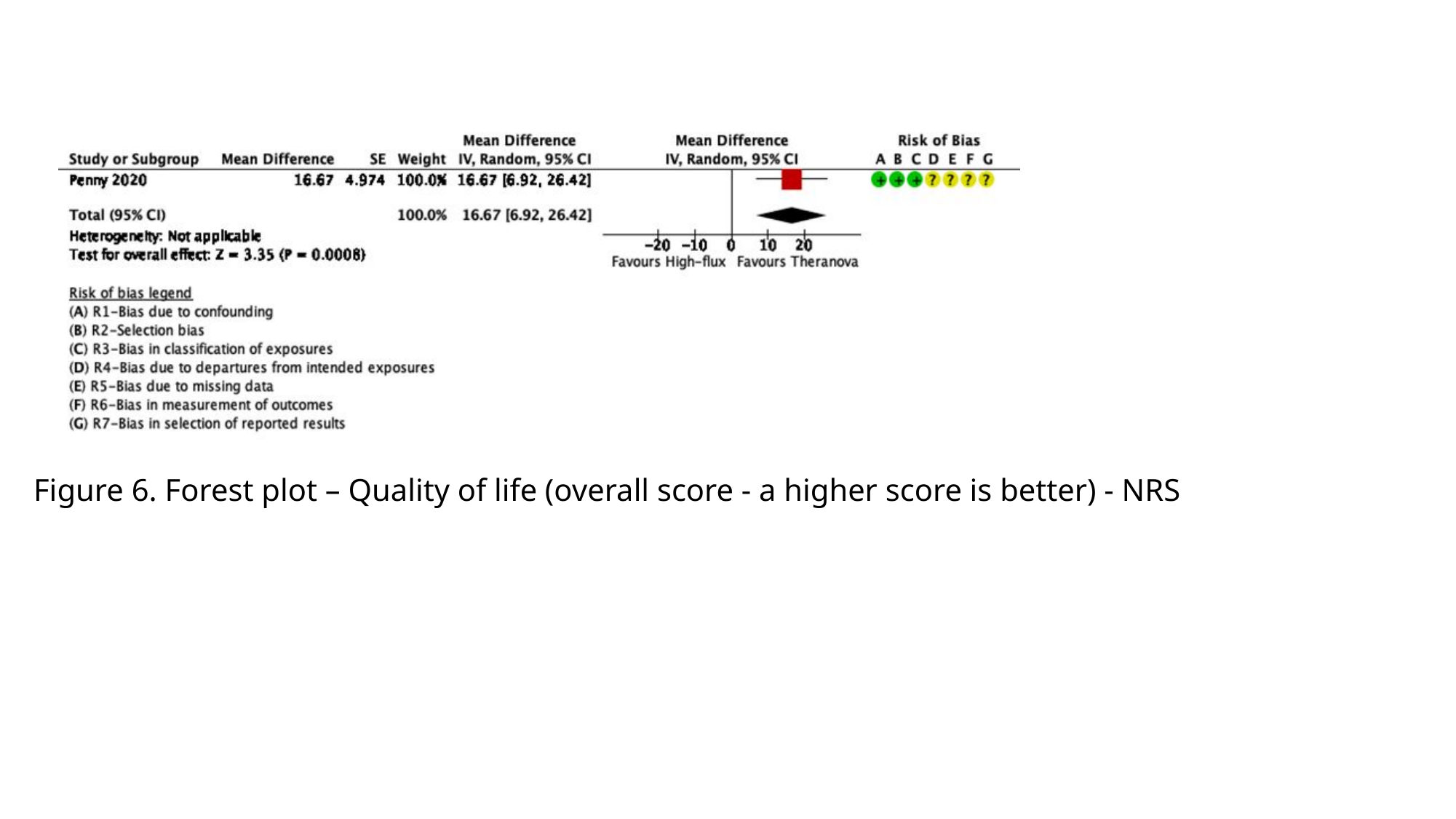

Figure 6. Forest plot – Quality of life (overall score - a higher score is better) - NRS

## Slide 7
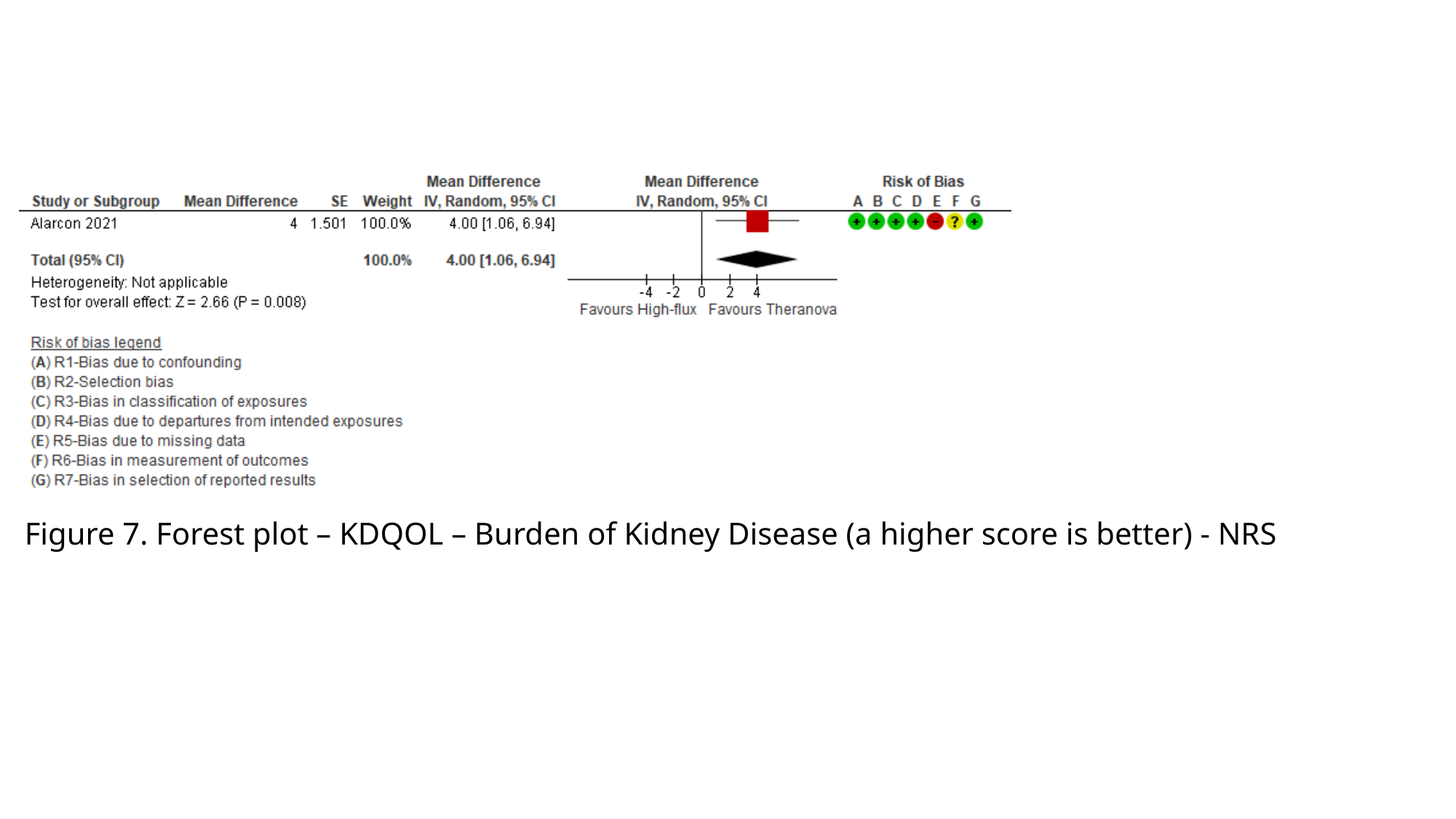

Figure 7. Forest plot – KDQOL – Burden of Kidney Disease (a higher score is better) - NRS

## Slide 8
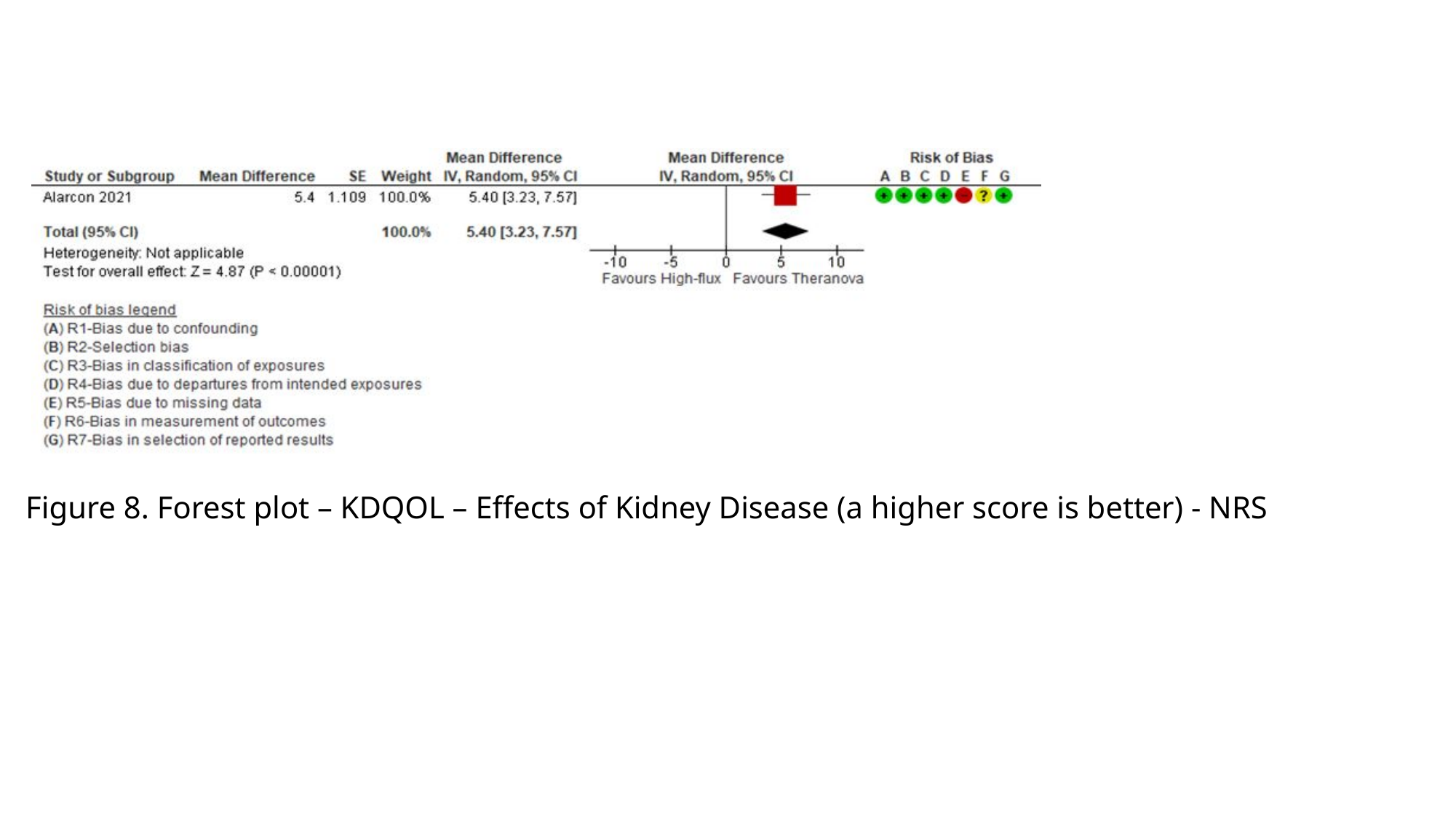

Figure 8. Forest plot – KDQOL – Effects of Kidney Disease (a higher score is better) - NRS

## Slide 9
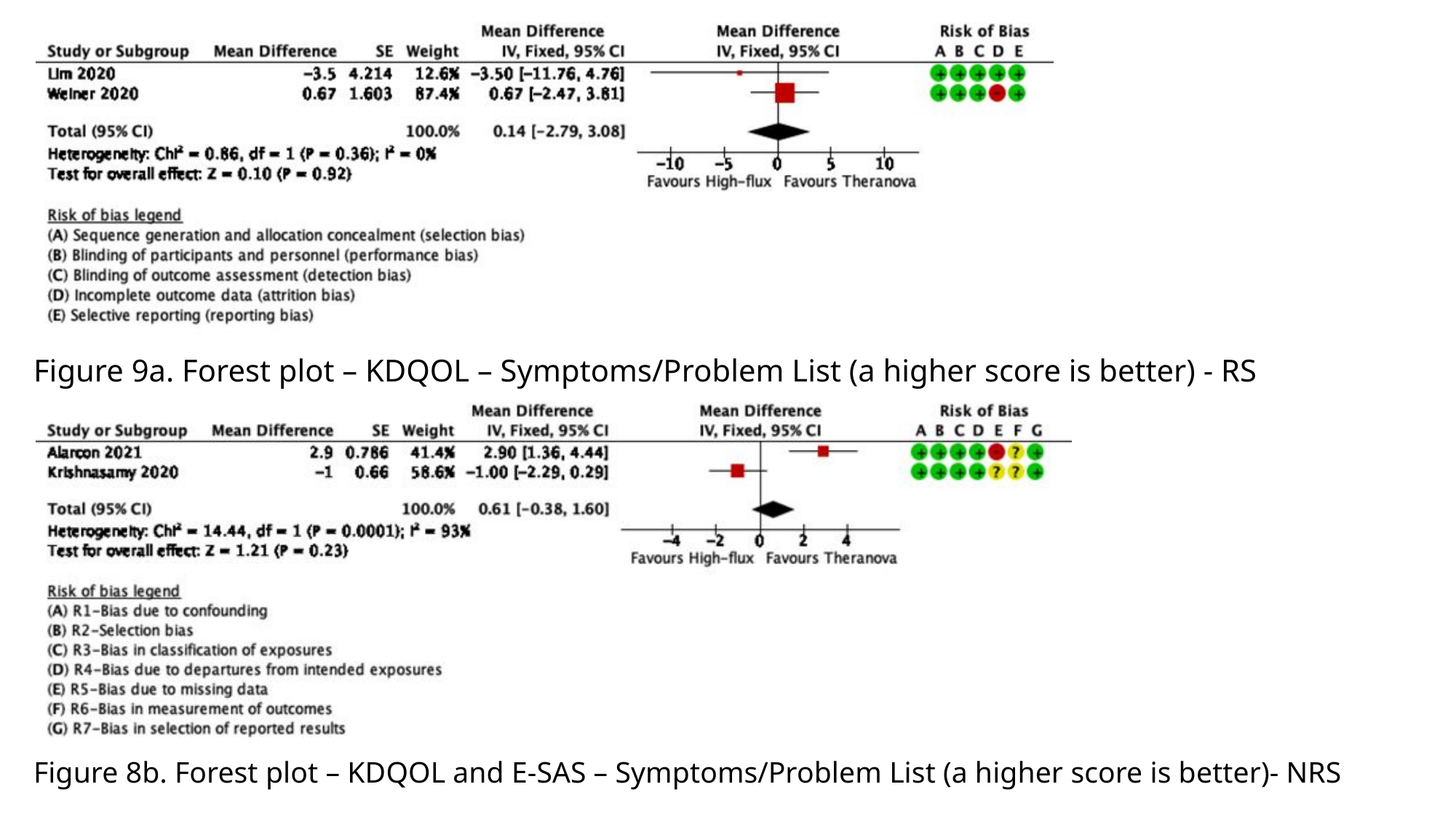

Figure 9a. Forest plot – KDQOL – Symptoms/Problem List (a higher score is better) - RS
Figure 8b. Forest plot – KDQOL and E-SAS – Symptoms/Problem List (a higher score is better)- NRS

## Slide 10
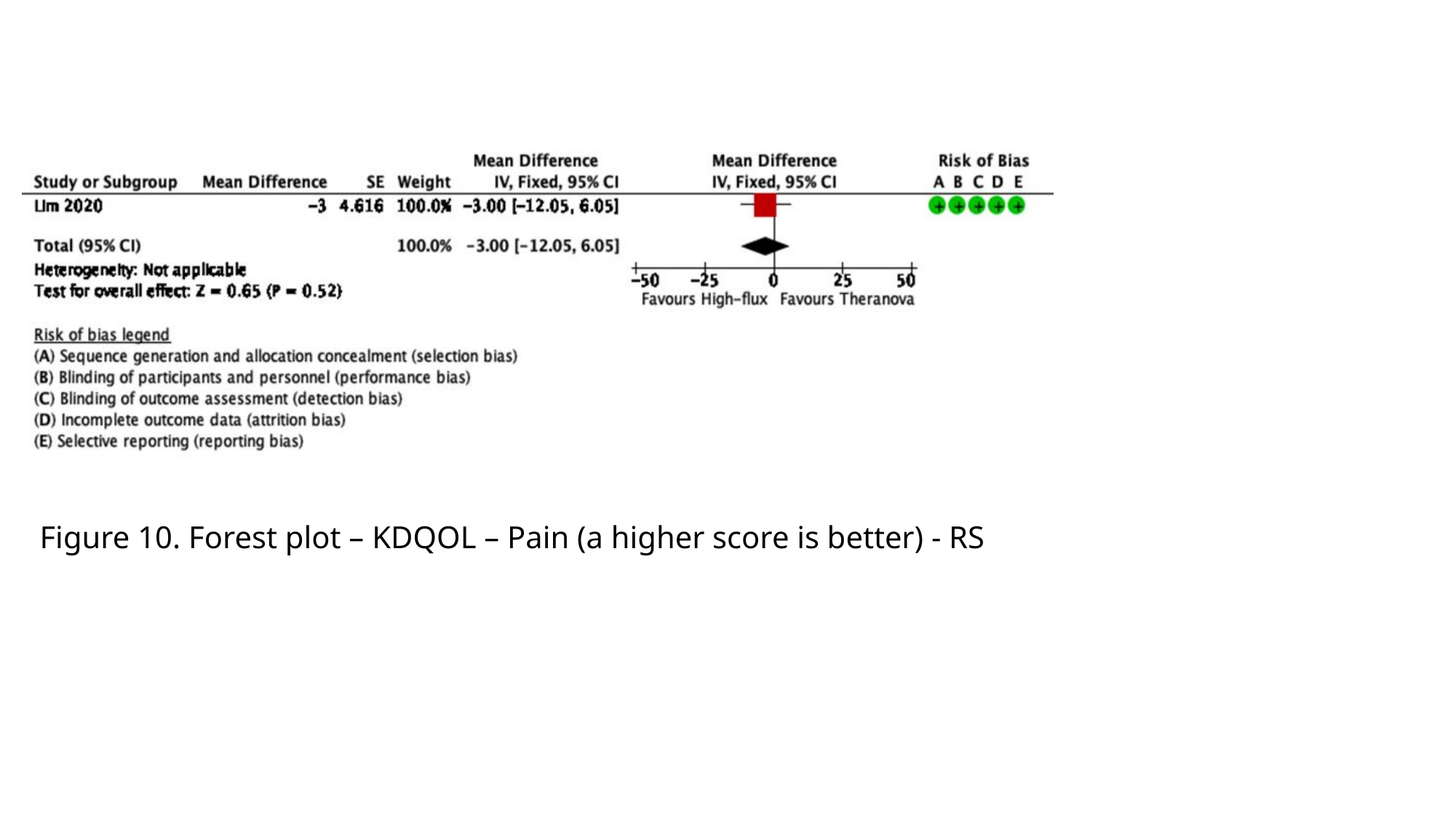

# Figure 10. Forest plot – KDQOL – Pain (a higher score is better) - RS

## Slide 11
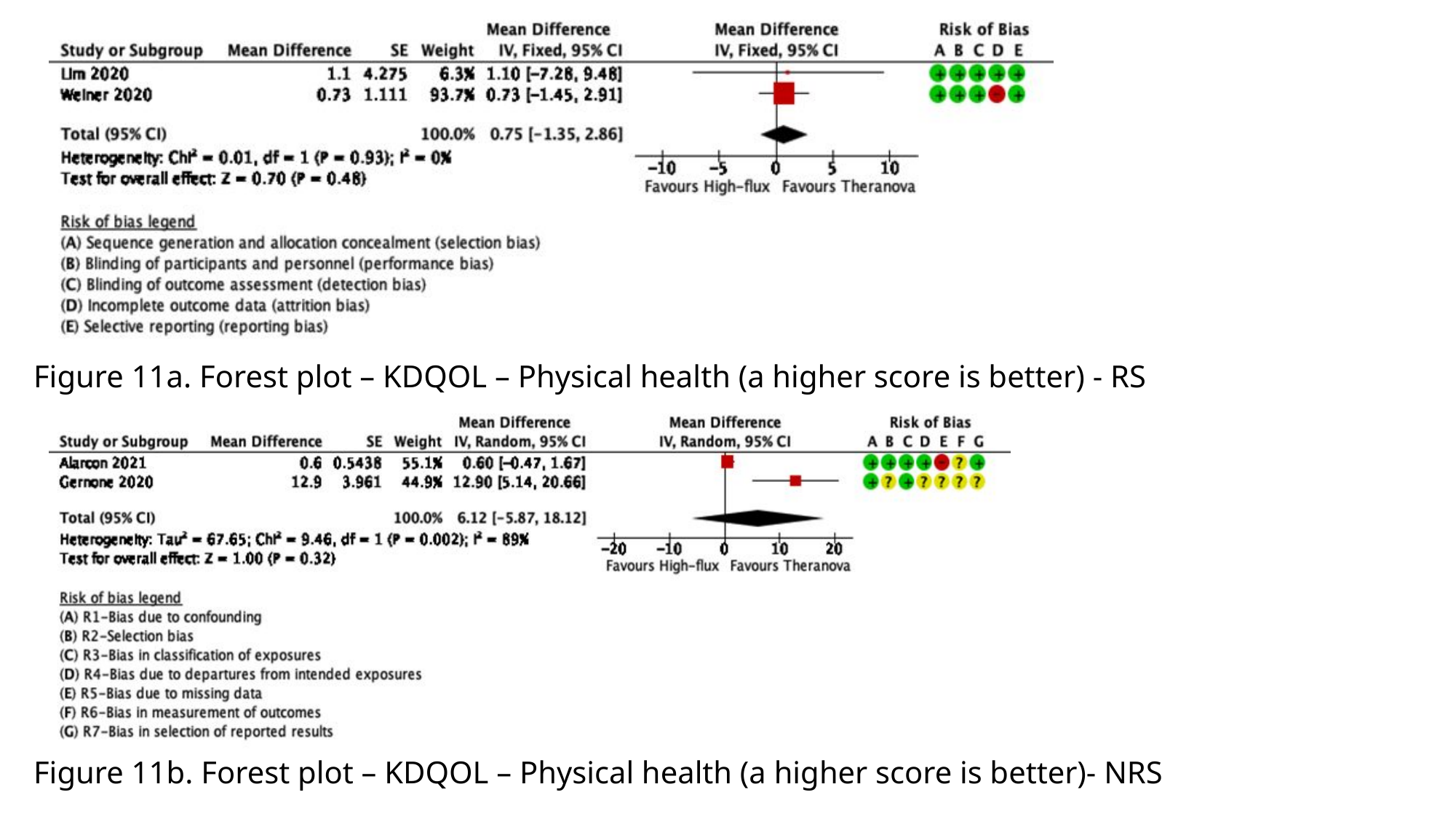

Figure 11a. Forest plot – KDQOL – Physical health (a higher score is better) - RS
Figure 11b. Forest plot – KDQOL – Physical health (a higher score is better)- NRS

## Slide 12
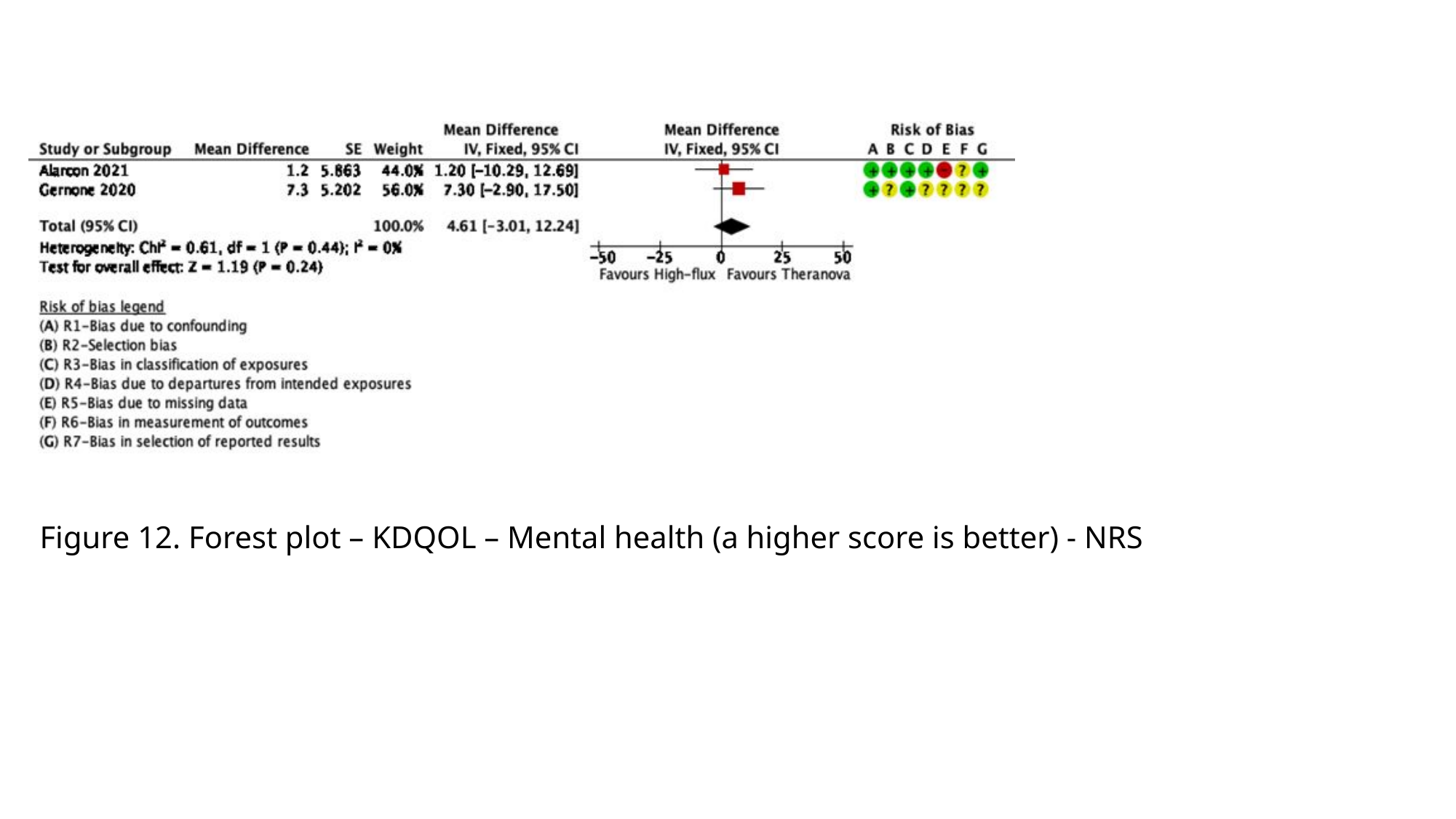

# Figure 12. Forest plot – KDQOL – Mental health (a higher score is better) - NRS

## Slide 13
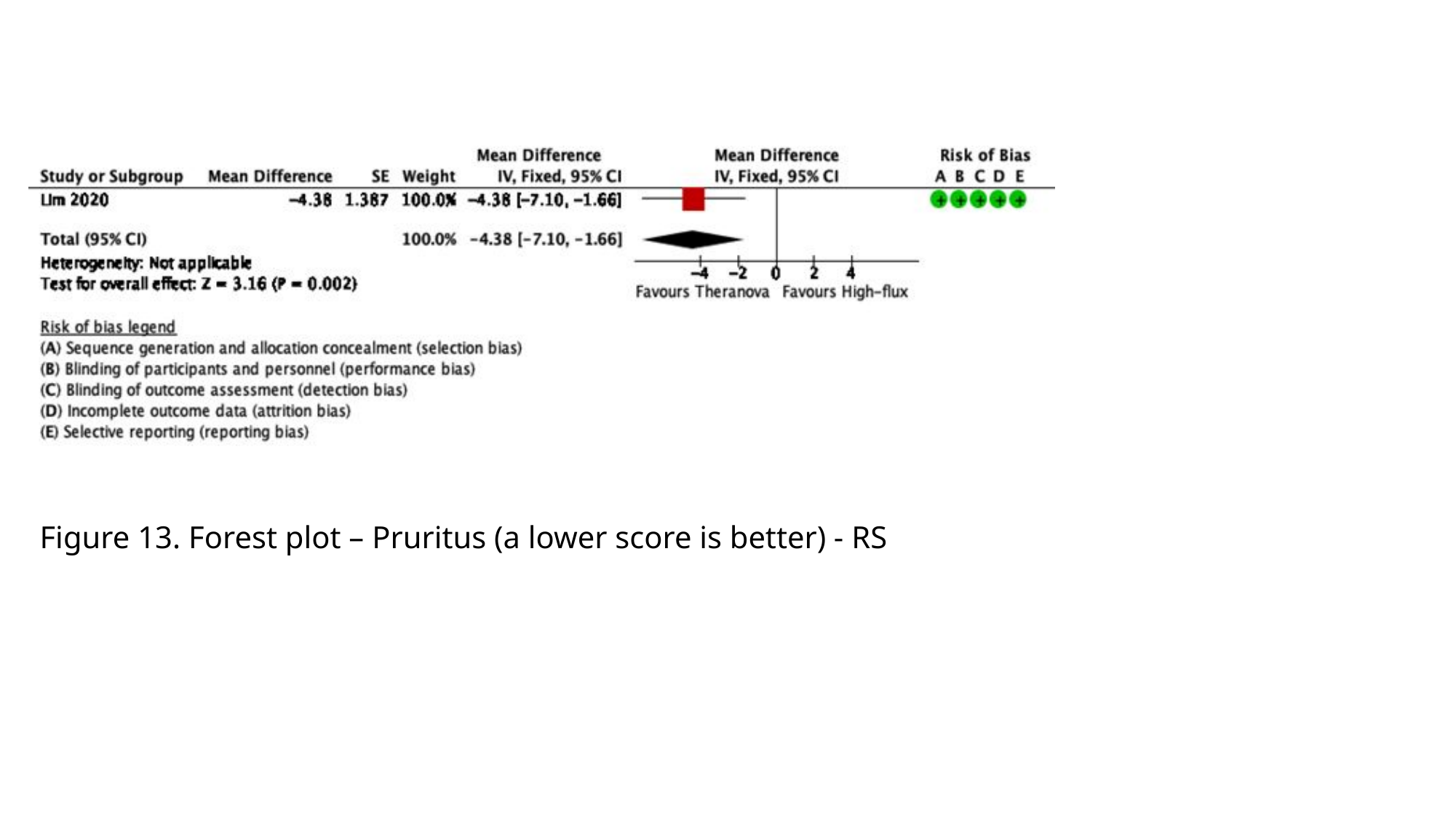

# Figure 13. Forest plot – Pruritus (a lower score is better) - RS

## Slide 14
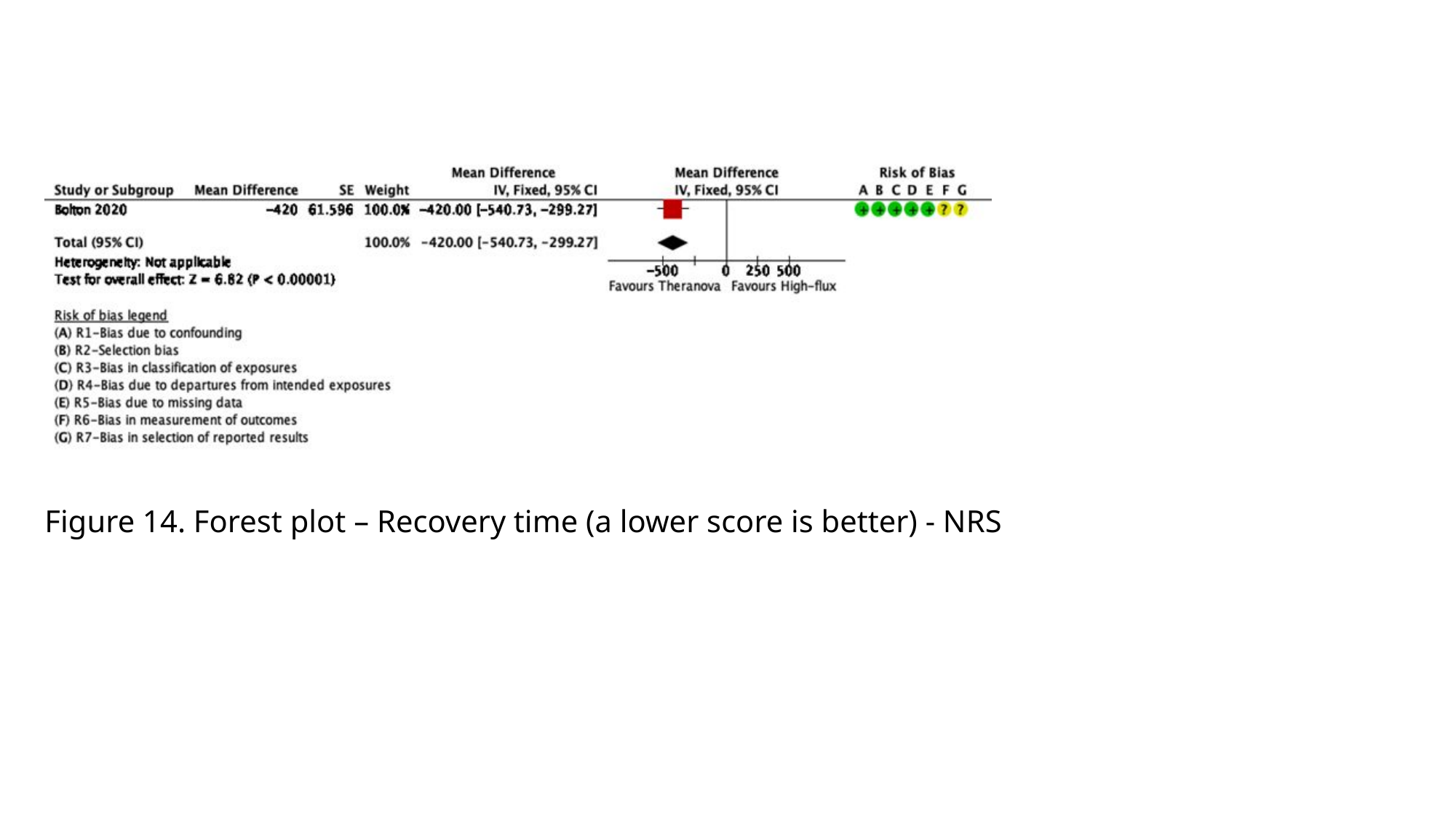

# Figure 14. Forest plot – Recovery time (a lower score is better) - NRS

## Slide 15
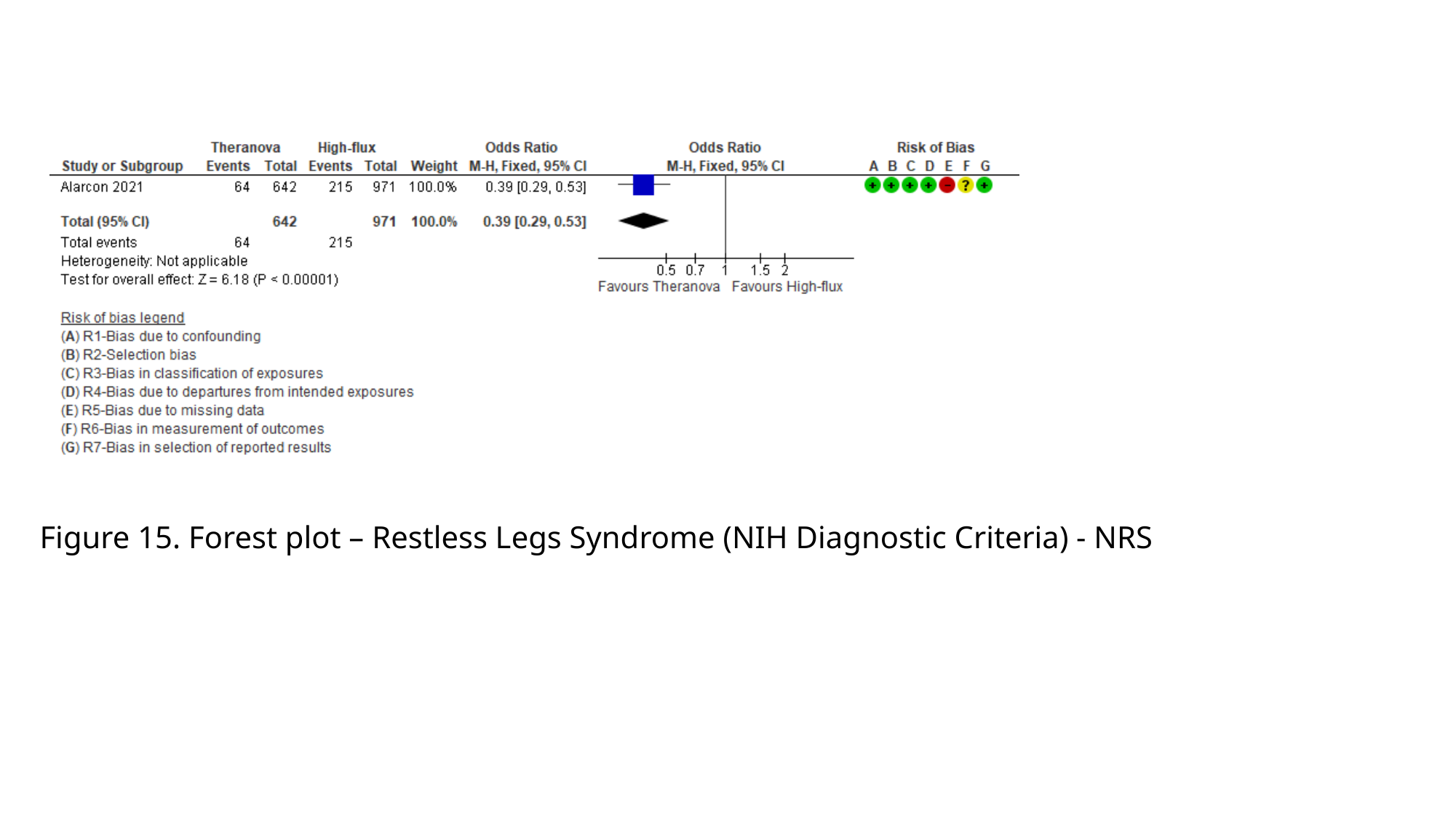

# Figure 15. Forest plot – Restless Legs Syndrome (NIH Diagnostic Criteria) - NRS

## Slide 16
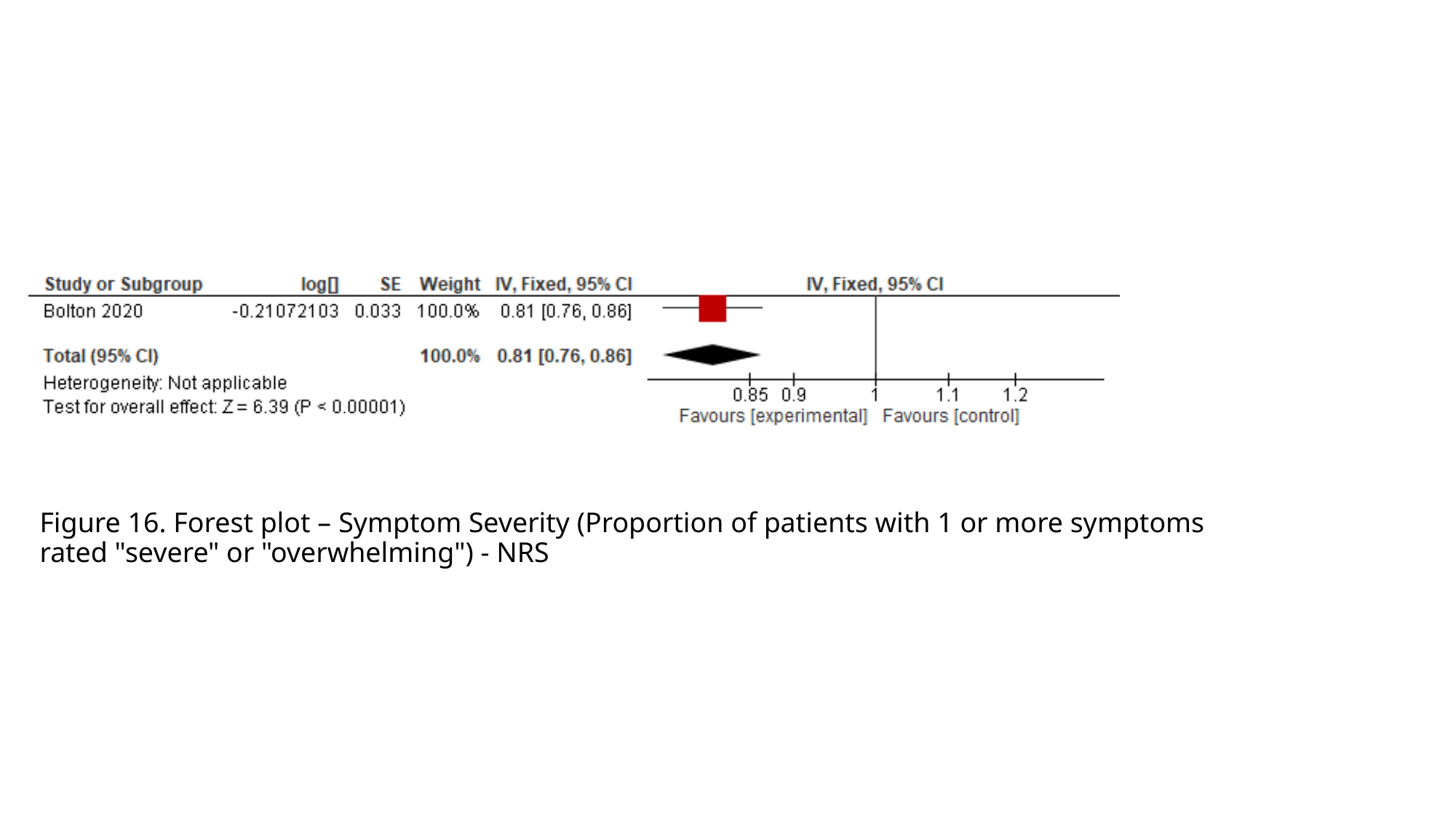

# Figure 16. Forest plot – Symptom Severity (Proportion of patients with 1 or more symptoms rated "severe" or "overwhelming") - NRS

## Slide 17
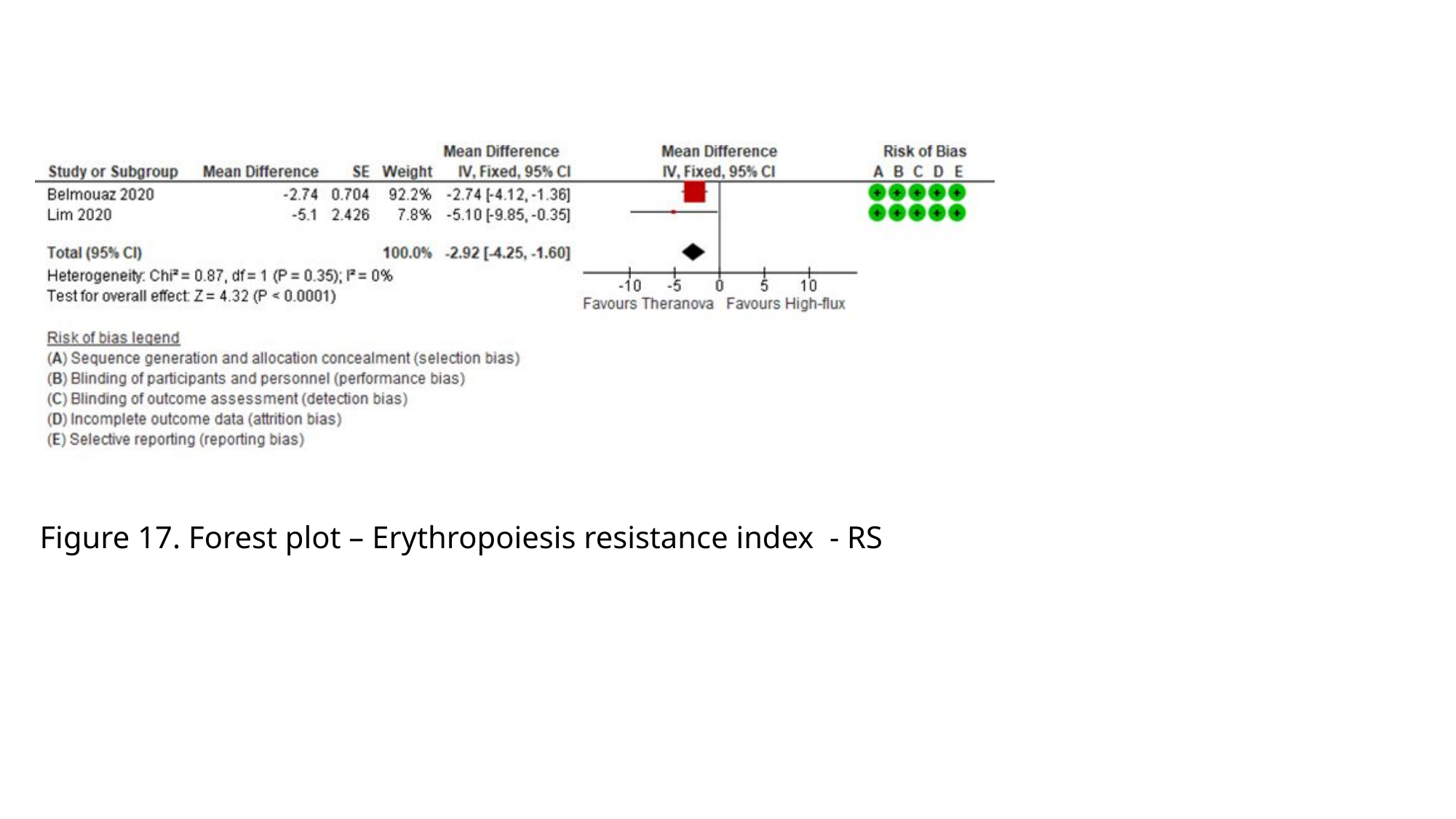

# Figure 17. Forest plot – Erythropoiesis resistance index  - RS

## Slide 18
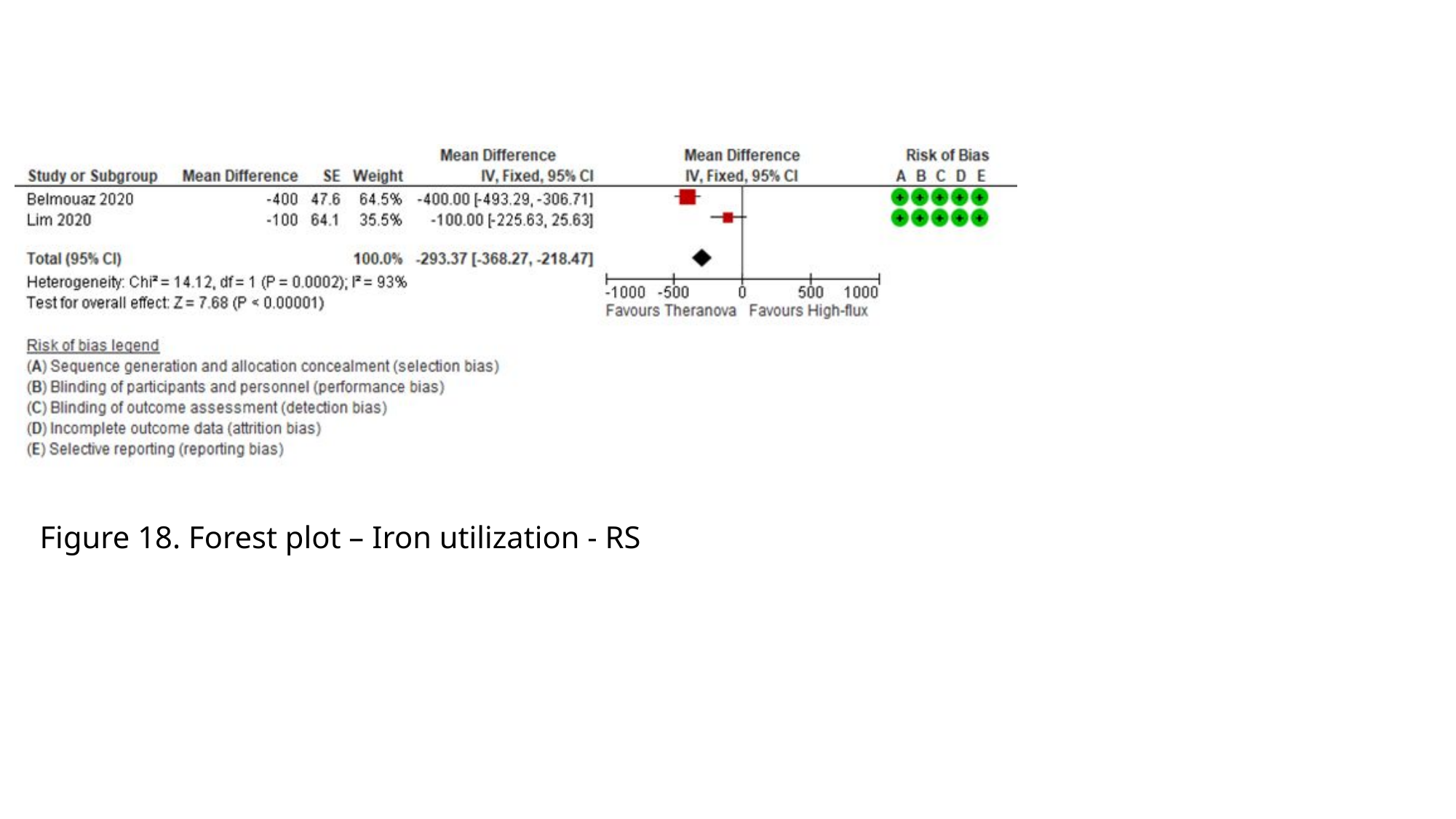

# Figure 18. Forest plot – Iron utilization - RS
