# Supplementary material for: Clinical Outcomes With Medium Cut-Off Versus High-Flux Hemodialysis Membranes: A Systematic Review and Meta-Analysis
Source: Can J Kidney Health Dis. 2022 Jan 21;9:20543581211067087. doi: 10.1177/20543581211067087 (PMC8785433; doi:10.1177/20543581211067087)
Supplement: sj-docx-7-cjk-10.1177_20543581211067087 – Supplemental material for Clinical Outcomes With Medium Cut-Off Versus High-Flux Hemodialysis Membranes: A Systematic Review and Meta-Analysis [file sj-docx-7-cjk-10.1177_20543581211067087.docx]

| Appendix G – Complete Summary of Findings Table (detailed explanations for certainty ratings in footnotes) | | | | | | |
| --- | --- | --- | --- | --- | --- | --- |
| **Theranova compared to High Flux Membranes for ESRD** | | | | | | |
| **Patient or population**: ESRD  **Setting**: Maintenance hemodialysis  **Intervention**: Theranova  **Comparison**: High Flux Membranes | | | | | | |
| Outcome № of participants  (studies) | Relative effect (95% CI) | **Anticipated absolute effects (95% CI)** | | | Certainty | What happens |
|  |  |  |  | **Difference** |  |  |
| All-cause mortality assessed with: number of deaths from any cause follow up: range 12 weeks to 52 weeks № of participants: 306 (4 RCTs) | **RR 0.93** (0.31 to 2.78) | 3.1% | **2.9%** (1 to 8.6) | **0.2% fewer** (2.1 fewer to 5.5 more) | ⨁⨁◯◯ LOW ^a^ | Theranova may result in little to no difference in survival. |
| All-cause mortality assessed with: number of deaths from any cause follow up: range 26 weeks to 52 weeks № of participants: 166 (4 observational studies) | **RR 0.85** (0.12 to 5.91) | 2.1% | **1.8%** (0.3 to 12.4) | **0.3% fewer** (1.9 fewer to 10.3 more) | ⨁⨁◯◯ LOW ^a^ | Theranova may result in little to no difference in survival. |

| All-cause mortality assessed with: number of deaths from any cause follow up: range 12 weeks to 52 weeks № of participants: 597 (4 RCTs and 4 observational Studies) | not estimable | 2.1% | **1.6%** | **0.4% fewer** (2.8 fewer to 2.1 more) | ⨁⨁⨁◯ MODERATE ^c^ | Theranova likely results in little to no difference in survival. |
| --- | --- | --- | --- | --- | --- | --- |

| Hospitalization assessed with: number of episodes of hospitalization for any reason follow up: 24 weeks № of participants: 172 (1 RCT) | **Rate ratio 0.48** (0.27 to 0.84) | 43.0% | **20.7%** (11.6 to 36.1) | **22.4% fewer** (31.4 fewer to 6.9 fewer) | ⨁⨁◯◯ LOW ^b,c^ | Theranova may result in a reduction in hospitalization. |
| --- | --- | --- | --- | --- | --- | --- |
| Hospitalization assessed with: number of episodes of hospitalization for any reason follow up: range 26 weeks to 52 weeks № of participants: 102 (2 observational studies) | **Rate ratio 0.39** (0.06 to 2.37) | 59.4% | **23.2%** (3.6 to 140.8) | **36.2% fewer** (55.8 fewer to 81.4 more) | ⨁◯◯◯ VERY LOW ^c,d,e^ | The evidence is very uncertain about the effect of Theranova on hospitalization. |
| Hospitalization length of stay (LOS) assessed with: number of days in hospital follow up: 52 weeks № of participants: 81 (1 observational study) | - | The mean hospitalization length of stay was **5.94** days | - | MD **1.5 days lower** (2.22 lower to 0.78 lower) | ⨁⨁⨁◯ MODERATE ^d^ | Theranova likely reduces hospitalization days. |
| Serious adverse events (SAE) assessed with: death or any life threatening condition leading to hospitalization follow up: range 12 weeks to 26 weeks № of participants: 312 (4 RCTs) | **Rate ratio 0.63** (0.38 to 1.04) | 23.6% | **14.9%** (9 to 24.5) | **8.7% fewer** (14.6 fewer to 0.9 more) | ⨁⨁◯◯ LOW ^b,c^ | Theranova may result in little to no difference in SAE's. |
| Infection assessed with: number of infections requiring treatment follow up: range 24 weeks to 26 weeks № of participants: 113 (2 observational studies) | **Rate ratio 0.38** (0.17 to 0.85) | 17.2% | **6.5%** (2.9 to 14.6) | **10.6% fewer** (14.3 fewer to 2.6 fewer) | ⨁⨁⨁◯ MODERATE ^c,f^ | Theranova likely reduces infection. |
| Infection assessed with: number of infections requiring treatment follow up: range 24 weeks to 26 weeks № of participants: 113 (2 observational studies) | **RR 0.53** (0.29 to 0.99) | 17.2% | **9.1%** (5 to 17) | **8.1% fewer** (12.2 fewer to 0.2 fewer) | ⨁⨁⨁◯ MODERATE ^c,f^ | Theranova likely reduces infection. |
| Quality of life assessed with: KDQOL-SF-36 and EQ-5D; a higher score is better № of participants: 222 (2 RCTs) | - | The mean quality of life ranged from **51-72** | - | MD **2.18 higher** (3.29 lower to 7.65 higher) | ⨁⨁◯◯ LOW ^b,g^ | Theranova may result in little to no difference in quality of life. |
| Quality of life assessed with: London Evaluation of Illness (LEVIL); a higher score is better follow up: 12 weeks № of participants: 28 (1 observational study) | - | The mean quality of life was **51** points | - | MD **16.67 points higher** (6.92 higher to 26.42 higher) | ⨁⨁⨁◯ MODERATE ^b,h,i^ | Theranova likely results in a large increase in quality of life among individuals with low (<70/100) baseline scores. |
| Burden of kidney disease  assessed with: KDQOL; a higher score is better follow up: range 12 weeks to 24 weeks № of participants: 222 (2 RCTs) | - | The mean burden of kidney disease ranged from **31-37** | - | MD **3.5 lower** (8.5 lower to 1.51 higher) | ⨁⨁◯◯ LOW ^b,c^ | Theranova may result in little to no difference in burden of kidney disease scores. |
| Burden of kidney disease assessed with: KDQOL; a higher score is better follow up: 52 weeks № of participants: 992 (1 observational study) | - | The mean burden of kidney disease was **46** points | - | MD **4 points higher** (1.06 higher to 6.94 higher) | ⨁⨁⨁◯ MODERATE ^j^ | Theranova likely results in a large increase in burden of kidney disease scores. |
| Effects of kidney disease assessed with: KDQOL; a higher score is better follow up: range 12 weeks to 24 weeks № of participants: 222 (2 RCTs) | - | The mean effects of kidney disease ranged from **68-77** | - | MD **1.88 lower** (5.23 lower to 1.47 higher) | ⨁⨁◯◯ LOW ^b,g^ | Theranova may result in little to no difference in effects of kidney disease scores. |
| Effects of kidney disease assessed with: KDQOL; a higher score is better follow up: 52 weeks № of participants: 992 (1 observational study) | - | The mean effects of kidney disease was **70** points | - | MD **5.4 points higher** (3.23 higher to 7.57 higher) | ⨁⨁⨁◯ MODERATE ^j^ | Theranova likely results in a large increase in effects of kidney disease scores. |
| Symptoms/problem list assessed with: KDQOL; a higher score is better follow up: range 12 weeks to 24 weeks № of participants: 222 (2 RCTs) | - | The mean symptoms/problem list ranged from **70-81** | - | MD **0.14 higher** (2.79 lower to 3.08 higher) | ⨁⨁◯◯ LOW ^b,g^ | Theranova may result in little to no difference in symptoms/problem list scores. |
| Symptoms/problem list  assessed with: KDQOL and E-SAS; a higher score is better follow up: range 24 weeks to 52 weeks № of participants: 1081 (2 observational studies) | - | The mean symptoms/problem list ranged from **79-89** | - | MD **0.61 higher** (0.38 lower to 1.6 higher) | ⨁⨁◯◯ LOW ^b,k^ | Theranova may result in little to no difference in kDQOL - Symptoms/Problem List. |
| Pain  assessed with: KDQOL; a higher score is better follow up: 12 weeks № of participants: 50 (1 RCT) | - | The mean pain was **69.3** points | - | MD **3 points lower** (12.05 lower to 6.05 higher) | ⨁⨁◯◯ LOW ^l^ | Theranova may result in little to no difference in pain scores. |
| Physical health assessed with: KDQOL - Physical Component Summary; a higher score is better follow up: range 12 weeks to 24 weeks № of participants: 222 (2 RCTs) | - | The mean physical health ranged from **44-52** | - | MD **0.75 higher** (1.35 lower to 2.86 higher) | ⨁⨁◯◯ LOW ^b,g^ | Theranova may result in little to no difference in physical health. |
| Physical health assessed with: KDQOL - Physical Component Summary; a higher score is better follow up: range 24 weeks to 52 weeks № of participants: 1003 (2 observational studies) | - | The mean physical health ranged from **27-41** | - | MD **6.12 higher** (5.87 lower to 18.12 higher) | ⨁⨁◯◯ LOW ^b,m^ | Theranova may result in little to no difference in physical health. |
| Mental health assessed with: KDQOL - Mental Composite Summary; a higher score is better follow up: range 12 weeks to 24 weeks № of participants: 222 (2 RCTs) | - | The mean mental health ranged from **44-51** | - | MD **3.18 higher** (5.65 lower to 12.01 higher) | ⨁⨁◯◯ LOW ^b,g^ | Theranova may result in little to no difference in mental health. |
| Mental health assessed with: KDQOL - Mental Composite Summary; a higher score is better № of participants: 1003 (2 observational studies) | - | The mean mental health ranged from **44-51** | - | MD **4.61 higher** (3.01 lower to 12.24 higher) | ⨁⨁⨁◯ MODERATE ^b^ | Theranova likely results in little to no difference in mental health. |
| Pruritus  assessed with: multidimensional pruritus questionnaire; a lower score is better follow up: 12 weeks № of participants: 50 (1 RCT) | - | The mean pruritus was **9.92** | - | MD **4.38 lower** (7.1 lower to 1.66 lower) | ⨁⨁⨁◯ MODERATE ^n^ | Theranova likely reduces pruritus. |
| Pruritus (additional analysis based on visual-analog scale - VAS) assessed with: VAS; ; a lower score is better follow up: 12 weeks № of participants: 50 (1 RCT) | - | The mean pruritus (additional analysis based on visual-analog scale - VAS) was **0** | - | MD **1.18 lower** (2.05 lower to 0.31 lower) | ⨁⨁⨁◯ MODERATE ^n^ | Theranova likely reduces pruritus based on visual-analog scale (VAS). |
| Recovery time assessed with: minutes to recover questionnaire; intention-to-treat analysis; a lower score is better follow up: 52 weeks № of participants: 89 (1 observational study) | - | The mean recovery time was **600** Minutes | - | MD **420 Minutes lower** (541 lower to 299 lower) | ⨁⨁⨁⨁ HIGH ^o^ | Theranova results in large reduction in minutes to recover after dialysis. |
| Recovery time assessed with: Minutes to recover questionnaire; per protocol analysis; a lower score is better follow up: 52 weeks № of participants: 89 (1 observational study) | - | The mean recovery time was **273** minutes | - | MD **177 minutes lower** (298.7 lower to 56.4 lower) | ⨁⨁⨁⨁ HIGH ^o^ | Theranova results in large reduction in minutes to recover after dialysis. |
| Restless Legs Syndrome - NRS assessed with: NIH Diagnostic Criteria № of participants: 992 (1 observational study) | **OR 0.39** (0.29 to 0.53) | 22.1% | **10.0%** (7.6 to 13.1) | **12.2% fewer** (14.5 fewer to 9 fewer) | ⨁⨁⨁◯ MODERATE ^b^ | Theranova likely results in a large reduction in restless legs syndrome. |
| Symptom Severity - NRS  assessed with: Palliative Care Outcome Scale - Symptom Module (Proportion of patients with 1 or more symptoms rated "severe" or "overwhelming") follow up: 52 weeks № of participants: 89 (1 observational study) | **OR 0.81** (0.76 to 0.86) | 66.1% | **61.2%** (59.7 to 62.6) | **4.9% fewer** (6.4 fewer to 3.5 fewer) | ⨁⨁⨁◯ MODERATE ^n,p^ | Theranova likely reduces symptom severity. |
| Erythropoiesis resistance index follow up: 12 weeks № of participants: 90 (2 RCTs) | - | The mean erythropoiesis resistance index was **15** U/kg/week/g/L acheived Hb | - | **2.92 U/kg/week/g/L acheived Hb lower** (4.25 lower to 1.6 lower) | ⨁⨁⨁◯ MODERATE ^c^ | Theranova likely reduces erythropoiesis resistance index. |
| Erythropeiesis resistance index follow up: range 12 weeks to 52 weeks № of participants: 300 (6 observational studies) | - | - | - | SMD **0.67 SD lower** (1.29 lower to 0.04 lower) | ⨁⨁◯◯ LOW ^b,q^ | Theranova may reduce erythropeiesis resistance index. |
| Iron utilization assessed with: cumulative intravenous dose in 12 weeks (mg) follow up: 12 weeks № of participants: 90 (2 RCTs) | - | The mean iron utilization ranged from **700-1000** mg | - | MD **293 mg lower** (368 lower to 218 lower) | ⨁⨁⨁◯ MODERATE ^c^ | Theranova likely reduces iron utilization. |
| Iron utilization follow up: range 26 weeks to 52 weeks № of participants: 128 (2 observational studies) | - | - | - | SMD **0.13 SD lower** (0.42 lower to 0.15 higher) | ⨁⨁◯◯ LOW ^b,c^ | Theranova may result in little to no difference in iron utilization. |
| ***The risk in the intervention group** (and its 95% confidence interval) is based on the assumed risk in the comparison group and the **relative effect** of the intervention (and its 95% CI).   **CI:** Confidence interval; **RR:** Risk ratio; **MD:** Mean difference; **OR:** Odds ratio; **SMD:** Standardised mean difference | | | | | | |
| **GRADE Working Group grades of evidence** **High certainty:** We are very confident that the true effect lies close to that of the estimate of the effect **Moderate certainty:** We are moderately confident in the effect estimate: The true effect is likely to be close to the estimate of the effect, but there is a possibility that it is substantially different **Low certainty:** Our confidence in the effect estimate is limited: The true effect may be substantially different from the estimate of the effect **Very low certainty:** We have very little confidence in the effect estimate: The true effect is likely to be substantially different from the estimate of effect | | | | | | |

#### Explanations

a. Total event count < 10 across study populations, does not meet optimal information size criterion.

b. A high rate of attrition may have introduced serious risk of bias.

c. Small overall sample size; optimal information size criterion not met.

d. Arizia 2021 (N=81) was the larger among the studies reporting hospitalization outcomes and used a before-after design in which patients were enrolled only if they had a full year of follow-up before and after switching to Theranova. This design is high RoB due to selection and survivor bias, regression to the mean, and maturation/effect of time.

e. I2=93%; confidence intervals do not overlap.

f. Effect estimate is based on 2 NRS with before-after design and excludes 1 NRS (cohort design) which is higher RoB due to case-mix differences without statistical adjustment.

g. In rating the certainty that there is a treatment effect (threshold: null effect), the confidence interval crosses this threshold; OIS criterion not met.

h. Estimate is based on a subgroup of patients with low baseline QoL scores and results may not be applicable to patients with normal or high baseline scores.

i. QoL scores increased in a linear fashion over time during treatment with Theranova, then decreased linearly after crossing over to high-flux membranes; consistent with a dose-response effect; however, since the study has potential bias due to attrition, we did not rate up for dose-response or large effects.

j. The high rate of attrition introduces risk of survivor bias on patient-reported outcome measures in the large observational study.

k. I2=93%. In the study by Krishnasamy 2020, patients in the control group had scores of 89/100, resulting in a ceiling effect, contributing to inconsistency across studies.

l. Confidence interval includes appreciable benefit and harm (using 5 scalar units as the minimal important difference for KDQOL subscales); rated down two levels for imprecision.

m. I2=89%; confidence intervals do not overlap.

n. Small overall sample size in a single study; optimal information size criterion not met.

o. Although the study was prone to survivor bias due to attrition, a per-protocol analysis confirmed the magnitude and direction of effect observed in the ITT analysis; hence, we did not rate down for RoB.

p. Although this single study had significant attrition, this outcome was based on a per-protocol analysis that we considered low risk of bias, and we therefore did not rate down.

q. I2=87%
